# Extensive diversity and impact of drug-resistant HIV-1 variants in individuals with prior virologic failure

**DOI:** 10.1371/journal.ppat.1014118

**Published:** 2026-05-12

**Authors:** Melendhran Pillay, Manish C. Choudhary, Rinki Deo, Serhiy Naumenko, Trevor J. Tamura, Behzad Etemad, Gregory Edelstein, Aabida Khan, Kerusha Govender, Subitha Govender, Philip Tzou, Mary Mick, Zachary T. Herbert, Winnie Muyindike, Mahomed-Yunus Suleman Moosa, Jay Brijkumar, Henry Sunpath, Suzanne McCluskey, Benjamin Chimukangara, Sontaga C. Manyana, Lilishia Gounder, Raveen Parboosing, Kerri-Lee Francois, Robert Shafer, Shannan Ho Sui, Selvan Pillay, Vincent Marconi, Mark J. Siedner, Pravi Moodley, Nokukhanya Msomi, Jonathan Z. Li

**Affiliations:** 1 Department of Virology, National Health Laboratory Service, Inkosi Albert Luthuli Central Hospital, Durban, South Africa; 2 School of Laboratory Medicine and Medical Science, University of KwaZulu-Natal, Durban, South Africa; 3 Department of Medicine, Division of Infectious Diseases, Brigham and Women’s Hospital, Harvard Medical School, Boston, Massachusetts, United States of America; 4 Department of Biostatistics, Harvard Chan School of Public Health, Boston, Massachusetts, United States of America; 5 Newborn Screening Ontario, Ottawa, Ontario, Canada; 6 Department of Medicine, Division of Infectious Diseases, Stanford University, Stanford, California, United States of America; 7 Molecular Biology Core Facilities, Dana-Farber Cancer Institute, Boston, Massachusetts, United States of America; 8 Mbarara University of Science and Technology, Mbarara, Uganda; 9 Department of Infectious Disease, Nelson R. Mandela School of Medicine, University of KwaZulu-Natal, Durban, South Africa; 10 Massachusetts General Hospital and Harvard Medical School, Boston, Massachusetts, United States of America; 11 Division of Virology, National Health Laboratory Service & Faculty of Health Sciences, University of the Witwatersrand, Johannesburg, South Africa; 12 Department of Medicine, Division of Infectious Diseases, Stanford University, Stanford, California, United States of America; 13 Adrenergy Research Innovations, KwaZulu-Natal, South Africa; 14 Emory University School of Medicine and Rollins School of Public Health, Atlanta, GeorgiaUnited States of America; 15 Africa Health Research Institute, KwaZulu-Natal, South Africa; National Cancer Institute, UNITED STATES OF AMERICA

## Abstract

HIV-1 drug resistance remains a major challenge to treatment and control efforts, particularly in sub-Saharan Africa (sSA). However, standard resistance genotyping does not adequately capture linked drug resistance mutations within the viral quasispecies that may influence virologic failure (VF). We used a next-generation sequencing primer ID (NGS-Primer ID) assay to characterize linked HIV-1 drug resistance mutations in plasma from participants in the Resistance Testing to Improve Management of Virologic Failure (REVAMP) study who had detectable viremia on first-line non-nucleoside reverse transcriptase inhibitor (NNRTI)-based antiretroviral therapy (ART) and were maintained on NNRTI-based regimens. For each participant, we calculated a weighted genotypic susceptibility score (wGSS) based on the GSS of each reported pattern and its sequence-supported frequency within the sample. Plasma specimens from 108 participants were sequenced. Sanger sequencing showed a median GSS of 1.0 (IQR, 1.0-2.0), whereas NGS-Primer ID identified a median of 10 distinct resistance patterns per participant (IQR, 5–17), with 67% (IQR, 8–92) of reported DRM-pattern frequency within participants corresponding to linked dual-class mutations. A broad spectrum of drug-susceptible and resistant- DRM patterns were observed, with the median difference between the highest and lowest pattern-specific GSS being 2.0 (2.0-2.75). Within each participant, a median of 10% of reported patterns showed less resistance, while 19% showed more resistance compared to that predicted by Sanger sequencing. Individuals with persistent VF had significantly lower wGSS at study entry than those who later achieved virologic re-suppression (median, 1.3 vs 2.1; p < 0.001). NGS-Primer ID revealed substantial intra-host diversity in linked HIV-1 drug resistance patterns that was not captured by conventional Sanger sequencing. Incorporating linked resistance patterns into susceptibility assessment may improve prediction of subsequent virologic failure.

## Author summary

HIV-1 drug resistance remains a major challenge to treatment, especially in sSA, where treatment options may be limited. The clinical impact of linked resistance mutations on the same viral genome remains poorly understood because standard sequencing methods cannot fully resolve them. We used an NGS-Primer ID assay to detect linked HIV drug resistance mutations in people with HIV-1 subtype C who were failing a first-line non-nucleoside reverse transcriptase inhibitor (NNRTI)-based regimen in KwaZulu-Natal, South Africa. We found extensive diversity in drug resistance patterns both within and across individuals that was not captured by Sanger sequencing. We also found that a weighted genotypic susceptibility score based on linked variants at study entry differed significantly between individuals who later achieved virologic suppression and those with persistent virologic failure.

## Introduction

The World Health Organization estimated that more than 40.8 million people were living with HIV in 2024, with nearly two-thirds residing in sub-Saharan Africa (sSA) [1,2]. South Africa remains heavily affected by the HIV epidemic, particularly in KwaZulu-Natal (KZN), which has one of the highest adult HIV prevalences in the country [3,4]. Although South Africa has made major gains in ART scale-up, with over 5.9 million individuals receiving treatment by the end of 2023, ongoing virologic failure and increasing drug resistance continue to threaten these gains [2,5–7]. In response to rising NNRTI resistance, South Africa transitioned in 2019 to tenofovir/lamivudine/dolutegravir (TLD) as the preferred first- and second-line regimen [8].

HIV-1 drug resistance remains a major barrier to durable virologic suppression [9–12]. Because HIV replicates rapidly and with low polymerase fidelity, it exists within an individual as a genetically diverse viral quasispecies [13–15]. This within-host diversity may influence treatment outcome, particularly when drug-resistant minority variants (DRMVs), defined here as variants comprising <20% of the viral population, are present [16]. However, the clinical significance of DRMVs has been inconsistent across studies, with data from sub-Saharan Africa differing from findings in the United States and Europe [17–20]. Differences in viral subtype, adherence, and treatment context may contribute to these discrepancies [21,22].

Beyond the presence of individual drug resistance mutations, the genomic linkage of resistance mutations may also be clinically important. Prior studies suggest that virologic failure is more likely when mutations conferring resistance to more than one drug class are linked on the same viral genome than when they are present on separate variants [23–27]. However, data on linked dual-class resistance and its clinical relevance remain limited, particularly in African populations.

Standard Sanger sequencing does not reliably detect low-frequency variants and cannot determine whether resistance mutations are linked on the same viral genome, while conventional next-generation sequencing improves sensitivity but does not fully resolve linkage [28,29]. To address this limitation, we used an NGS-Primer ID assay, which labels individual cDNA molecules prior to amplification and reduces PCR-associated error and recombination [23,25,27]. We applied this approach to participants in the REVAMP trial with virologic failure on first-line non-nucleoside reverse transcriptase inhibitor (NNRTI)-based ART to characterize linked HIV-1 drug resistance patterns and assess their association with subsequent treatment outcome.

## Results

### Participant characteristics

A total of 108 participants failing a first-line NNRTI-based regimen consisting of TDF, FTC and EFV were included in this analysis (Table 1). Of the 108, 52% were female (52%), and the median age was 38 years (interquartile range [IQR] 31–44). The median baseline HIV-1 viral load at the time of VF was 4.1 log_10_ RNA copies/mL (IQR 3.6-4.8), with a median Sanger sequencing genotypic susceptibility score (GSS) of 1.0 (IQR 1.0-2.0). All participants had subtype C virus as determined by the Stanford HIV Drug Resistance Database, with phylogenetic analysis providing supportive evidence (S1 Fig).

**Table 1:** Participant characteristics at time of REVAMP study enrolment.

Characteristics	Value (n = 108)
Male *n* (%)	52 (48%)
Female *n* (%)	56 (52%)
Age in years, median (IQR)	38 (31–44)
Log_10_ viral load copies/mL, median (IQR)	4.1 (3.6-4.8)
CD4 count (cells/mm^3^), median (IQR)	284 (109-435)
Sanger sequencing GSS, median (IQR)	1.0 (1.0-2.0)
ART regimen: TDF, FTC, EFV (%)	108 (100%)

IQR, Interquartile range; mL, milliliter; CD4, cluster of differentiation 4; mm3, cubic millimeter, GSS, genotypic susceptibility scores; ART, antiretroviral therapy; TDF, tenofovir; FTC, emtricitabine; EFV, efavirenz.

### NGS-Primer ID uncovers remarkable diversity of HIV drug-resistance patterns

**Fig 1:**
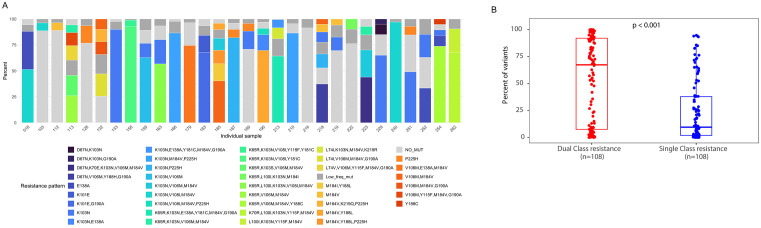
Distribution of HIV-1 mutational patterns among participants in the REVAMP trial. **(A)** A representative subgroup of participants is shown. Each stacked bar plot corresponds to an individual participant, and the colors represent specific mutational patterns. Pattern frequencies were calculated from the number of sequences assigned to each pattern relative to the total number of input sequences in the sample. Reported DRM patterns present at frequencies ≤5% are shown in dark grey. **(B)** Box plots showing the proportion of reported DRM-pattern frequency within participants with either dual (NRTI & NNRTI) or single drug class resistance mutations. Each dot represents a participant, the horizontal line within a box represents the median, the box represents the interquartile range, and the whiskers represent the data range excluding outliers. VF = virologic failure, VS = virologic suppression.

In this study, NGS-Primer ID was used to characterize the diversity of reported DRM patterns within each sample, which provides a far more detailed view of HIV drug resistance mutations than Sanger sequencing or traditional NGS, as illustrated in S2 Fig. NGS-Primer ID revealed a remarkable diversity of HIV-1 drug-resistance patterns, with a broad array of linked resistance mutations (Figs 1A; S3). The multiple colors within each stacked bar plot represent the relative proportions of reported DRM-containing and non-DRM patterns in each participant, including patterns harboring multiple linked resistance mutations. Reported DRM patterns present at frequencies ≤5% of total input sequences within a sample are grouped and shown in dark grey. Approximately one-third of participants harbored extreme diversity, with very low-frequency reported DRM patterns (each present at <5% of total input sequences within a sample) accounting for a substantial proportion of the reported patterns in some participants. A diverse range of mutational patterns as observed in Fig 1A were also observed across participants (S3 Fig). In this study, participants had a median of 10 [5, 17] different reported patterns, and 67% [8%, 92%] of the reported DRM-pattern frequency within an individual corresponded to dual-class resistance mutations (Fig 1B). Reported patterns with dual-class resistance mutations were present at significantly higher frequencies than reported DRM patterns harboring single-class resistance mutations (median 67.4% [IQR 7.5–92.0] vs 9.55% [IQR 1.9–37.9]; Wilcoxon rank-sum test p < 0.001; Fig 1B).

**Fig 2:**
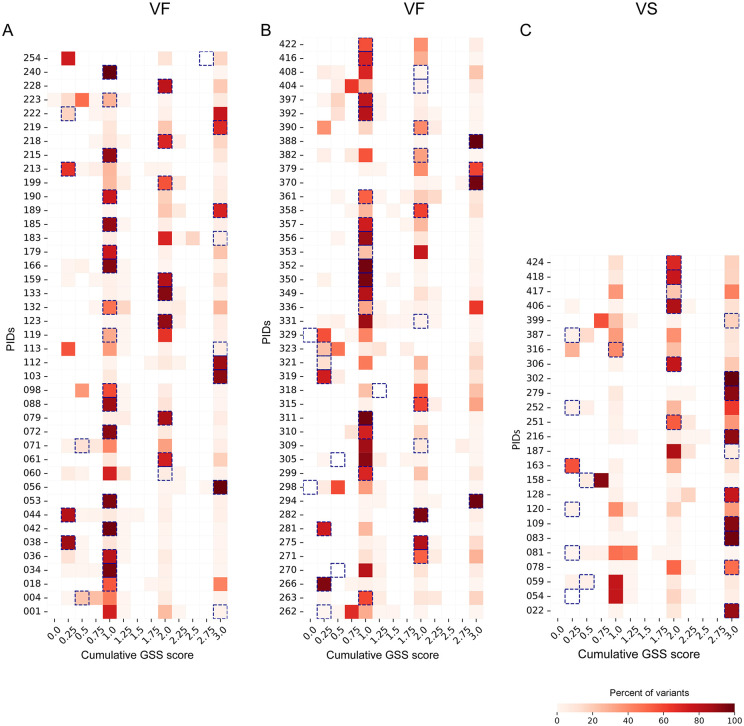
Distribution of reported pattern-level genotypic susceptibility scores within participants. Each row represents a unique study participant (y-axis) labeled with participant identification numbers (PIDs). The columns represent the cumulative genotypic susceptibility (GSS) values (x-axis), with lighter colors indicating the proportion of sequences assigned to patterns closer to 0%, and darker red shades represents closer to 100%. Sanger Sequencing GSS is denoted by dotted boxes. The first two heatmaps (A & B) show participants with virologic failure (VF) and the third heatmap (C) shows participants with virologic suppression (VS).

We calculated the genotypic susceptibility score (GSS) for each reported pattern and uncovered a diverse range of GSS values within each individual. This significant intra-host variation was not detected by conventional Sanger sequencing, highlighting the increased sensitivity of our single-genome resolution approach (Fig 2). We observed co-occurrence of both resistant and susceptible reported patterns within all 108 participants, as illustrated in Fig 2.

### Sanger sequencing can underestimate or overestimate the level of drug resistance present within the viral quasispecies

**Fig 3:**
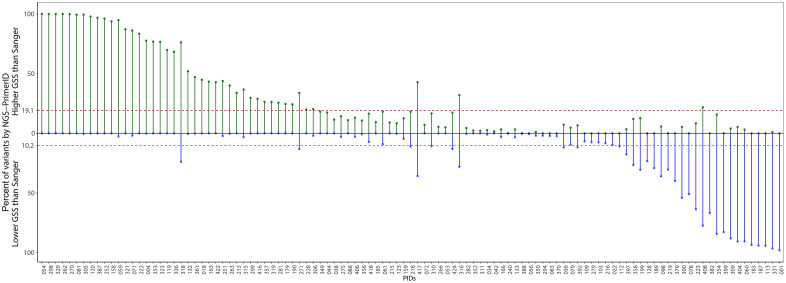
Proportion of reported patterns with discordant genotypic susceptibility scores as detected by NGS-Primer ID relative to Sanger sequencing. Each column (x-axis) represents a different participant as shown by unique participant identification numbers (PIDs), and the y-axis shows the proportion of sequences assigned to reported patterns by NGS-Primer ID that had a higher GSS (Green lollipops showing GSS > 0, indicating less resistance;) or lower GSS (Blue lollipops showing GSS < 0, indicating greater resistance) when compared to Sanger sequencing. The dashed lines represent the median proportion of variants identified by NGS-Primer ID with higher GSS than Sanger or lower GSS than Sanger.

We next examined variants for which GSS values differed between Sanger sequencing and NGS–Primer ID. Nearly 30% of all reported patterns showed discordant GSS values when assessed by NGS–Primer ID (Fig 3). At the participant level, NGS–Primer ID identified both increased and decreased levels of resistance compared with Sanger sequencing. Within each participant, a median of 10.2% of reported patterns exhibited higher GSS values (indicating greater drug susceptibility) than predicted by Sanger sequencing, whereas a median of 19.1% of reported patterns showed lower GSS values (indicating greater resistance). Across participants, there was substantial within-host heterogeneity, with a median range of pattern-specific GSS values (difference between the highest and lowest GSS) of 2.0 (IQR 2.0–2.75).

**Fig 4:**
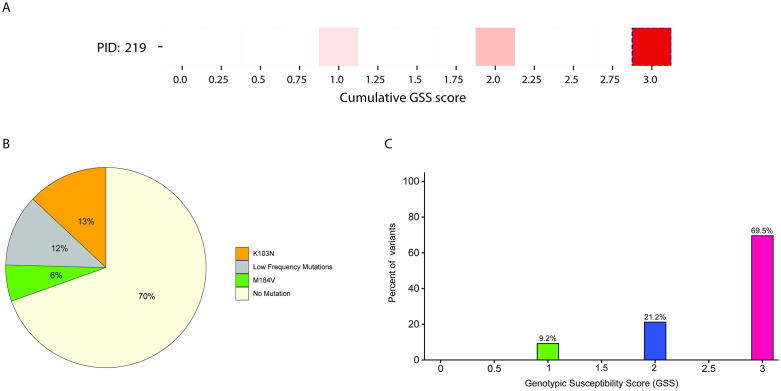
Reported pattern-level genotypic susceptibility scores and mutational patterns in participant 219. **(A)** Heatmap showing variant-level genotypic susceptibility scores (GSS) identified by NGS-Primer ID. The non-dashed boxes indicate the distribution of NGS-Primer ID-derived GSS values across variants in this participant. **(B)** Pie chart showing the distribution of mutational patterns detected in participant 219. **(C)** Histogram showing the distribution of reported patterns across GSS categories.

**Fig 5:**
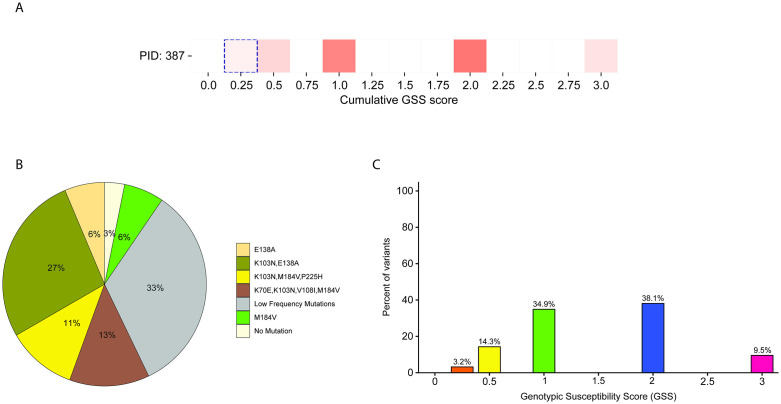
Reported pattern-level genotypic susceptibility scores and mutational patterns in participant 387. **(A)** Heatmap showing the distribution of variant-level genotypic susceptibility scores (GSS) identified by NGS-Primer ID, together with the corresponding Sanger sequencing GSS. **(B)** Pie chart showing the distribution of distinct mutational patterns detected in participant 387. **(C)** Histogram showing the distribution of reported patterns across GSS categories.

Representative data from two participants exemplify the diverse mutational patterns observed across the entire 108-participant cohort. Detailed analysis of these select individuals provides further evidence that Sanger sequencing often misrepresents the level of resistance, either underestimating (Fig 4) or overestimating (Fig 5) the actual viral susceptibility present within a clinical specimen. In PID 219, Sanger sequencing identified a genotypic susceptibility score (GSS) of 3, implying a fully active ART regimen (Fig 4A). In contrast, the NGS-Primer ID platform identified 14 distinct mutational patterns, including multiple variants possessing drug resistance (Figs 4B; S4). This high-resolution analysis confirmed that while 69.5% of variants remained fully susceptible with a GSS of 3, 30.4% of the viral variants demonstrated measurable drug resistance. This subset included 21.2% of variants with a GSS of 2 and 9.2% of variants harboring both K103N and M184V mutations (Fig 4B; 4C), resulting in a GSS of 1 for those specific viral genomes (Fig 4C). These findings illustrate the limitations of Sanger sequencing in resolving the true diversity and extent of resistant variants within a viral quasispecies. Furthermore, our data suggest that Sanger sequencing can misrepresent the population by either underestimating or overestimating the resistance burden present in the quasispecies.

For PID 387, Sanger sequencing yielded high-level resistance against all ARVs, leading to a GSS = 0.25 (Fig 5A). However, NGS-Primer ID identified extremely diverse mutational patterns (i.e., 18 different mutational patterns) present within the quasispecies of this participant (Figs 5B; S5), with only 3.2% of variants actually harboring resistance to all ARVs, i.e., GSS = 0.25 (Fig 5C). Approximately 83% of variants had a GSS ≥ 1 and 48% had a GSS > 2. Together, these results demonstrate that NGS-Primer ID provides a more granular view of the viral quasispecies and reveals how Sanger sequencing can underestimate or overestimate the levels of drug resistance present within an individual. The mutational patterns and GSS obtained from Sanger Sequencing for all participants are shown in S1 Table.

### Weighted genotypic susceptibility score by NGS-Primer ID distinguishes individuals with eventual virologic failure on continued NNRTI-based ART

**Fig 6:**
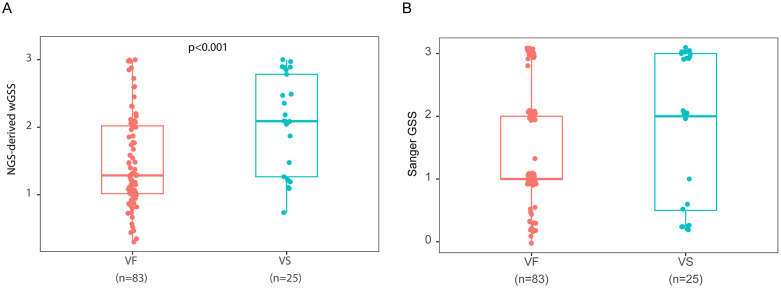
Weighted and Sanger-derived genotypic susceptibility scores by subsequent virologic outcome. **(A)** Box plots comparing weighted genotypic susceptibility scores (wGSS) derived from NGS-Primer ID at study entry between participants with subsequent virologic failure (VF) and those with virologic suppression (VS). **(B)** Box plots comparing Sanger-derived genotypic susceptibility scores (GSS) at study entry between participants with subsequent virologic failure (VF) and those with virologic suppression (VS). In both panels, the center line indicates the median, the box indicates the interquartile range, and the whiskers indicate the data range excluding outliers. wGSS = weighted GSS; VS = Virologic Suppression; VF = Virologic Failure.

The weighted GSS (wGSS) was calculated by taking the average GSS across all reported patterns identified by NGS-Primer ID at the time of study entry to predict subsequent virologic suppression with continued NNRTI-based ART. Participants with persistent virologic failure (VF) had lower wGSS at study entry than those who achieved virologic suppression (median 1.3 [IQR 1.0–2.0] vs 2.1 [IQR 1.3–2.8]; Wilcoxon rank-sum test p < 0.001; Fig 6A). In contrast, the use of GSS by Sanger sequencing at the time of study entry did not demonstrate any significant differences between individuals who eventually had VF or re-suppression (Fig 6B).

## Discussion

In this study, NGS-Primer ID revealed substantial within-host diversity in linked HIV-1 drug resistance patterns among participants with virologic failure on first-line NNRTI-based ART. Compared with Sanger sequencing, this approach identified a much more complex mixture of susceptible and resistant variants within individual participants, including frequent dual-class linked resistance mutations. Variant-specific genotypic susceptibility scores varied widely within participants, and nearly 30% of variants showed GSS values that differed from those predicted by Sanger sequencing. Importantly, a weighted genotypic susceptibility score derived from linked variants at study entry distinguished participants with persistent virologic failure from those who later achieved virologic suppression, whereas Sanger-based GSS did not.

These findings help explain why standard resistance genotyping may not always reflect subsequent clinical outcome in individuals with virologic failure. Sanger sequencing provides a consensus-level view of the viral population and does not reliably detect low-frequency variants or determine whether resistance mutations are linked on the same viral genome. As a result, clinically relevant within-host heterogeneity may be missed, leading to an incomplete picture of overall drug susceptibility within a sample [28,30,31]. This interpretation is consistent with prior work showing that conventional resistance testing does not always align with treatment outcome, particularly in resource-limited settings and in individuals with complex resistance patterns [32–37]. In our cohort, this was evident in both directions, with Sanger sequencing underestimating resistance in some participants and overestimating it in others. The participant-level examples further show that consensus genotyping may not reflect the relative distribution of susceptible and resistant variants within a specimen.

Our results also extend previous work on DRMVs by showing that the genomic context in which resistance mutations occur may be as important as their presence alone. Studies examining the relationship between DRMVs and virologic failure have reported inconsistent findings across geographic and treatment settings [10,17,19–22,31,38,39]. One likely explanation is that assays focused only on the presence or frequency of individual mutations cannot determine whether mutations affecting more than one drug class are linked on the same viral genome. In our cohort, participants had a median of 10 distinct resistance patterns, and dual-class resistance variants were common and often present at higher frequencies than single-class resistant variants. Together, these findings suggest that the structure of the viral quasispecies, rather than simply the presence of isolated mutations, may contribute meaningfully to treatment outcome [24,26].

Our findings are also in line with prior studies showing that linked dual-class resistance mutations are associated with an increased risk of virologic failure [24,26]. By incorporating both variant-specific susceptibility and relative abundance, wGSS provides a more representative measure of the resistance landscape within a participant than consensus genotyping alone. In this study, lower wGSS values at study entry were associated with persistent virologic failure on continued NNRTI-based ART, suggesting that haplotype-informed susceptibility metrics may better identify individuals at risk of poor response when treatment is not changed.

The paper has several limitations. First, this analysis was performed on plasma specimens from participants receiving NNRTI-based regimens, which are no longer the dominant first-line therapy in many settings following the transition to dolutegravir-based treatment [8,40]. Even then, these findings find relevance; NNRTI-associated resistance continues to shape treatment histories in many ART-experienced individuals, rilpivirine remains a component of long-acting treatment strategies, and the broader principle that linked resistance patterns may improve prediction of treatment outcome is likely applicable beyond NNRTI-based regimens [41,42]. Second, this was a retrospective analysis based on stored specimens from a selected subset of REVAMP participants [37,43]. Because our sequencing was restricted to the time of study entry (TP1) to predict subsequent outcomes, we were unable to evaluate longitudinal changes, such as transient or persistent failure at subsequent time points. Third, although NGS-Primer ID reduces PCR-associated error and recombination and enables single-genome resolution, it is technically demanding and remains better suited to research settings than routine clinical implementation at present [23,25,27]. In addition, the ability to detect very low-frequency variants depended on sample-specific sequence support and was therefore not uniform across all specimens. Accordingly, variants near the 1% level should be interpreted cautiously. Finally, adherence and pharmacologic factors were not directly incorporated into this analysis, although both are likely to contribute importantly to subsequent virologic outcome [32,34].

Despite these limitations, this study provides proof-of-principle evidence that resolving linked HIV-1 resistance mutations can yield clinically meaningful information not captured by standard genotyping. In settings where treatment options are limited or where individuals remain on partially active regimens, a more accurate view of the within-host resistance landscape may improve risk stratification and inform regimen selection. More broadly, these findings support further evaluation of linkage-informed resistance approaches in contemporary ART settings, including INSTI-based regimens and long-acting therapies [41,44]. Furthermore, future longitudinal studies are needed to prospectively track how the composition and linkage structure of these complex variants shift under ongoing selective pressure.

In summary, NGS-Primer ID uncovered extensive within-host diversity in HIV-1 drug resistance patterns among participants with prior virologic failure on NNRTI-based ART and showed that a weighted susceptibility measure based on linked resistance patterns may better predict subsequent treatment outcome than Sanger sequencing. These findings underscore the importance of considering both the composition and linkage structure of the viral quasispecies when interpreting HIV drug resistance and provide a foundation for future studies in the setting of modern ART regimens.

## Methods

### Ethics Statement

This study was approved by the Mass General Brigham Institutional Review Board (IRB) and the Biomedical Research Ethics Committee (BREC) at the University of KwaZulu-Natal (BREC reference number: BREC/00005292/2023 and REVAMP BREC reference number: BFC377/16). This study did not obtain informed consent from individuals, due to use of anonymized stored specimens for genotyping therefore the need for informed consent was waived upon ethics approval.

### Study participants

Remnant plasma specimens were obtained from participants in the REVAMP (Resistance Testing Versus Adherence Monitoring for Patients) study conducted in KZN, South Africa. The REVAMP study was a randomized controlled study (2016–2019), aimed at investigating whether resistance testing improves rates of virologic re-suppression after VF, among individuals receiving HIV care in public health programs across sSA (NCT02787499) [37,43]. The REVAMP study had two arms: a standard of care (SOC) arm and a resistance testing (RT) arm. Participants were enrolled and randomized to each arm from four hospitals in the eThekwini District of KZN (Addington, Clairwood, King Dinizulu, and Wentworth). Additional details on the REVAMP study are described in the S1 Appendix. In this analysis, we included South African participants with VF who were continued on an NNRTI-based ART regimen with adherence counselling.

### Study specimens

We performed retrospective viral Sanger sequencing on stored frozen plasma specimens from participants enrolled in the REVAMP study [37,43]. Additional details on the selection of specimens for NGS-primer ID testing are described in the Supplementary appendix (S2-S5 Tables). A control library of clonal HIV-1 subtype C sequences was prepared at defined concentrations by mixing clones at concentrations of 68.4%, 25%, 5%, 1%, 0.5% and 0.1% and processed in parallel with the specimens [29]. The PCR amplicons from the control library were gel purified, quantified by Nanodrop spectrophotometry and used to validate our library preparation method, to delineate the error rate and to determine the threshold for detecting DRMVs down to 0.1% of the viral population. The presence of minority variants (MVs) present at nucleotide positions in the control library amplicon was assessed for having the same invariant base [29]. The upper range of the assay error rate quantified as 3 standard deviations (SDs) above the mean MV percentage detected among the invariant bases [29].

### Laboratory procedures for Sanger sequencing

Plasma specimens with viral loads ≥1000 copies/mL were retrieved from -80°C storage and equilibrated to room temperature before processing. HIV-1 viral RNA was extracted from 1mL of patient plasma specimens using a NucliSENS easyMAG automated extraction platform (BioMérieux, Marcy l’Etoile, France), according to manufacturer’s instructions. RNA was reverse-transcribed into cDNA using SuperScript III First-Strand Synthesis System (ThermoFisher Scientific, Massachusetts, USA) and HIV-1 subtype C gene-specific primers.

A nested PCR amplification of the protease and reverse transcriptase genes was performed using Southern African Treatment Resistance Network custom primers [45]. Cycle sequencing reactions were performed on purified products using a Big-Dye Terminator v3.1 kit (Applied Biosystems, Foster City, CA, USA). We assessed sequence quality and generated consensus sequence alignments using Geneious Prime software 2021.1.1 (Biomatters Ltd, New Zealand). We detected drug resistance mutations and determined GSS values for efavirenz (EFV), emtricitabine (FTC), and tenofovir disoproxil fumarate (TDF) using the Stanford University HIV Drug Resistance Database v9.0 [46].

### Primer ID–based reverse transcription and cDNA generation

HIV-1 viral RNA was extracted from stored remnant plasma specimens (viral loads ≥1000 copies/ml) using the TRIzol (ThermoFisher Scientific, Massachusetts, USA) method, according to manufacturer’s instructions. Extracted RNA was reverse transcribed using the SuperScript IV RT-PCR kit (ThermoFisher Scientific, Waltham, USA) consisting of a high-fidelity reverse transcriptase capable of synthesizing a 600 bp region of HIV-1 *pol* gene spanning the major primary and secondary resistance mutations for the *reverse transcriptase* gene (codons 38–239). The cDNA synthesis was performed in a 50μl reaction consisting of a 5μM concentration of HIV-1 subtype C specific primer with Primer ID tags (S6-S8 Tables), 10mM dNTPs (Thermofisher Scientific, USA), 5 × RT Buffer (Thermofisher Scientific, USA), 0.1M DTT, 100mM RNaseOUT (Thermofisher Scientific, USA; 40 U/μl), and SuperScript IV RT (Thermofisher Scientific, USA; 200 U/μl). The primers were degraded, and the RNA/DNA duplex was denatured with Ribonuclease H (RNase H) (5 units; New England Biolabs, USA). cDNA with primer ID-tags were precipitated overnight. S2 Fig and the S1 Appendix provide an overview and hypothetical example of how NGS-Primer ID offers a more detailed view of the diversity of resistance variants beyond that of Sanger or traditional NGS.

### PCR amplification, library construction, and next-generation sequencing

Primer ID-tagged cDNA molecules were amplified in 5 replicates using 2 × Kapa Hi-Fi Hot Start Uracil + reaction mix (KAPA Biosystems, Massachusetts, USA). A PCR master mix consisting of a concentration of 10μM forward primer 2589 FC in the first round PCR, 10μM forward primer 2709 FC in the second round, and a 10μM deoxyuridine (dU)-containing reverse primer PrimRegion- R-5Us in both rounds of PCR master mix was prepared as described in the S9-S11 Tables.

The five replicates from each PCR round were combined and purified using the QIAquick PCR Purification Kit (Qiagen, Hilden, Germany), according to manufacturer’s instructions. The PCR products enriched with dU primers were treated with uracil-DNA glycosylase (UDG) followed by cleavage at abasic sites, yielding double stranded DNA (dsDNA) with 17-nucleotide (nt) 3′-overhangs on both ends. NGS linkers were hybridised to the 17-nt 3′ overhangs and the dsDNA was completed with Klenow fragment DNA polymerase and dNTPs. The linker ligated DNA libraries were resolved on a 1.5% agarose gel and the ~ 719 bp fragment was excised and purified using QIAquick Gel Extraction kit (Qiagen, Hilden, Germany), according to manufacturer’s instructions. MiSeq Illumina sequencing was performed on primer ID tagged DNA libraries using the MiSeq v2 kit (Illumina Incorporation, California, USA). Sequence fastq files were demultiplexed and exported for bioinformatics analyses. The sequences were analyzed using the HIV-DRLink tool [47,48]. Additional details on the sequence analysis are described in the S1 Appendix.

### HIV Drug-Resistance Analysis Pipeline (hivdrm) and Statistical Analysis

Sequencing reads were quality filtered, demultiplexed, and trimmed to remove adapters, primers, barcodes, and UMI sequences. For UMI-tagged libraries, reads sharing the same UMI were grouped and collapsed into consensus sequences using an 80% nucleotide agreement threshold to reduce PCR and sequencing errors. Consensus sequences were aligned to the study reference sequence corresponding to the targeted genomic region, and linked haplotypes were reconstructed across the amplicon. Drug-resistance mutations were identified and interpreted using the hivdrm pipeline (available at https://github.com/rinkideo/hivdrm) together with SierraPy, a Python interface to the Stanford HIV Drug Resistance Database [49] that translates viral sequences into drug-resistance mutations and predicts susceptibility to individual antiretroviral drugs. The pipeline summarizes reported DRM patterns, the number of sequences supporting each DRM pattern, and the corresponding pattern frequencies within a sample. For each sample, the frequency of a given DRM pattern was calculated as the number of sequences assigned to that pattern divided by the total number of input sequences in the sample. Accordingly, the summed frequency of all reported DRM patterns reflects the proportion of sample sequences containing one or more reported DRMs, whereas the remainder represents sequences without reported DRMs. These summary metrics therefore reflect sequence-level support for reported DRM patterns and should not be interpreted as direct measures of total sample-level template sampling depth or total recovered UMI counts. More details are provided in the S1 Appendix.

We used non-parametric Wilcoxon rank sum test to compare the distribution of the wGSS between individuals with and without viral suppression on maintenance of first-line ART. We used version 1: R software (4.4.3) [50], tidyverse (2.0.0) [51] and ggplot2 (3.5.1) [52] for statistical analysis and figure plotting.

## Supporting information

S1 Fig Maximum likelihood phylogenetic tree. Maximum-likelihood phylogenetic tree including all sequences from participants in this study together with HIV-1 reference sequences in a rectangular layout. The tree was inferred using the generalized time reversible model with a proportion of invariant sites and gamma-distributed rate variation among sites (GTR + I + G). Branch support was assessed with 1,000 bootstrap replicates. Branch lengths represent the number of nucleotide substitutions per site (scale bar = 0.03).
(TIF)

S2 Fig Schematic illustration of genotypic susceptibility score (GSS) estimation from different sequencing approaches. The figure shows a hypothetical participant receiving an antiretroviral regimen consisting of efavirenz (EFV), tenofovir (TDF), and emtricitabine (FTC) (1). Example outputs are shown for Sanger sequencing (2), next-generation sequencing (NGS) (3), and NGS-based ultrasensitive single-genome sequencing with primer identifiers (NGS-PrimerID) (4). For each approach, detected drug resistance mutations are used to derive regimen-level GSS values. The schematic also illustrates summary GSS metrics derived from variant-level data, including maximum GSS, minimum GSS, and weighted GSS (5), and a conceptual distribution of GSS values across detected variants (6).
(TIF)

S3 Fig Distribution of viral mutational patterns across the study cohort. Each stacked bar plot corresponds to an individual participant, and the colors represent specific mutational patterns. Pattern frequencies were calculated from the number of sequences assigned to each pattern relative to the total number of input sequences in the sample. Reported DRM patterns present at frequencies ≤5% are shown in dark grey.
(TIF)

S4 Fig Detailed mapping of resistance patterns in PID 219. This figure illustrates a specific case where conventional Sanger sequencing underestimated the complexity of the reported resistance-pattern landscape in the sample. The first column identifies distinct mutational patterns, clarifying whether resistance markers are physically linked on individual viral genomes. The second and third columns quantify these patterns by listing the total number of sequences identified and their corresponding percentages within the specimen. Subsequent columns denote the presence of specific drug resistance mutations, which are highlighted with orange boxes. The final row provides the cumulative frequency of each individual mutation relative to the total number of input sequences in the sample.
(TIF)

S5 Fig Detailed mapping of resistance patterns in PID 387. This figure illustrates a specific case where conventional Sanger sequencing overestimated the complexity of the reported resistance-pattern landscape in the sample. The first column identifies distinct mutational patterns, clarifying whether resistance markers are physically linked on individual viral genomes. The second and third columns quantify these patterns by listing the total number of sequences identified and their corresponding percentages within the specimen. Subsequent columns denote the presence of specific drug resistance mutations, which are highlighted with orange boxes. The final row provides the cumulative frequency of each individual mutation relative to the total number of input sequences in the sample.
(TIF)

S1 Table Sample-level clinical, Sanger genotypic susceptibility, and NGS-Primer ID pattern summary metrics at HIV viral load time point 1. (DOCX)

S2 Table Genotypic Susceptibility Scores with corresponding susceptibility levels. (DOCX)

S3 Table Selection Criteria for Plasma specimens GSS ≥ 1. (DOCX)

S4 Table Selection criteria used for Plasma specimens with GSS < 1. (DOCX)

S5 Table Participant specimens selected from the REVAMP Study for NGS-Primer ID. (DOCX)

S6 Table cDNA Primers with Primer ID tags used for reverse transcription. (DOCX)

S7 Table Complimentary DNA synthesis Master Mix 1. (DOCX)

S8 Table Complimentary DNA synthesis Master Mix 2. (DOCX)

S9 Table First-Round Master Mix and Conditions. (DOCX)

S10 Table Second-Round Master Mix and Conditions. (DOCX)

S11 Table cDNA Amplification Primers used in First and Second Round PCR. (DOCX)

S1 Appendix Supplementary Appendix. Supplementary Methods: REVAMP Study: Participant enrolment and Randomization. REVAMP Study: Inclusion and Exclusion Criteria. Plasma Specimen Selection. Next-generation sequencing with Primer ID (NGS-Primer ID). HIV drug-resistance analysis pipeline (hivdrm). Phylogenetic Analysis. **Supplementary Results.** Diverse range of mutational patterns observed by NGS-Primer ID across all participants. Characterization of Resistant and Susceptible variants using weighted GSS by NGS-Primer ID.
(DOCX)
