# Supplementary figures and images for: Extensive diversity and impact of drug-resistant HIV-1 variants in individuals with prior virologic failure
Source: PLoS Pathog. 2026 May 12;22(5):e1014118. doi: 10.1371/journal.ppat.1014118 (PMC13221146; doi:10.1371/journal.ppat.1014118)

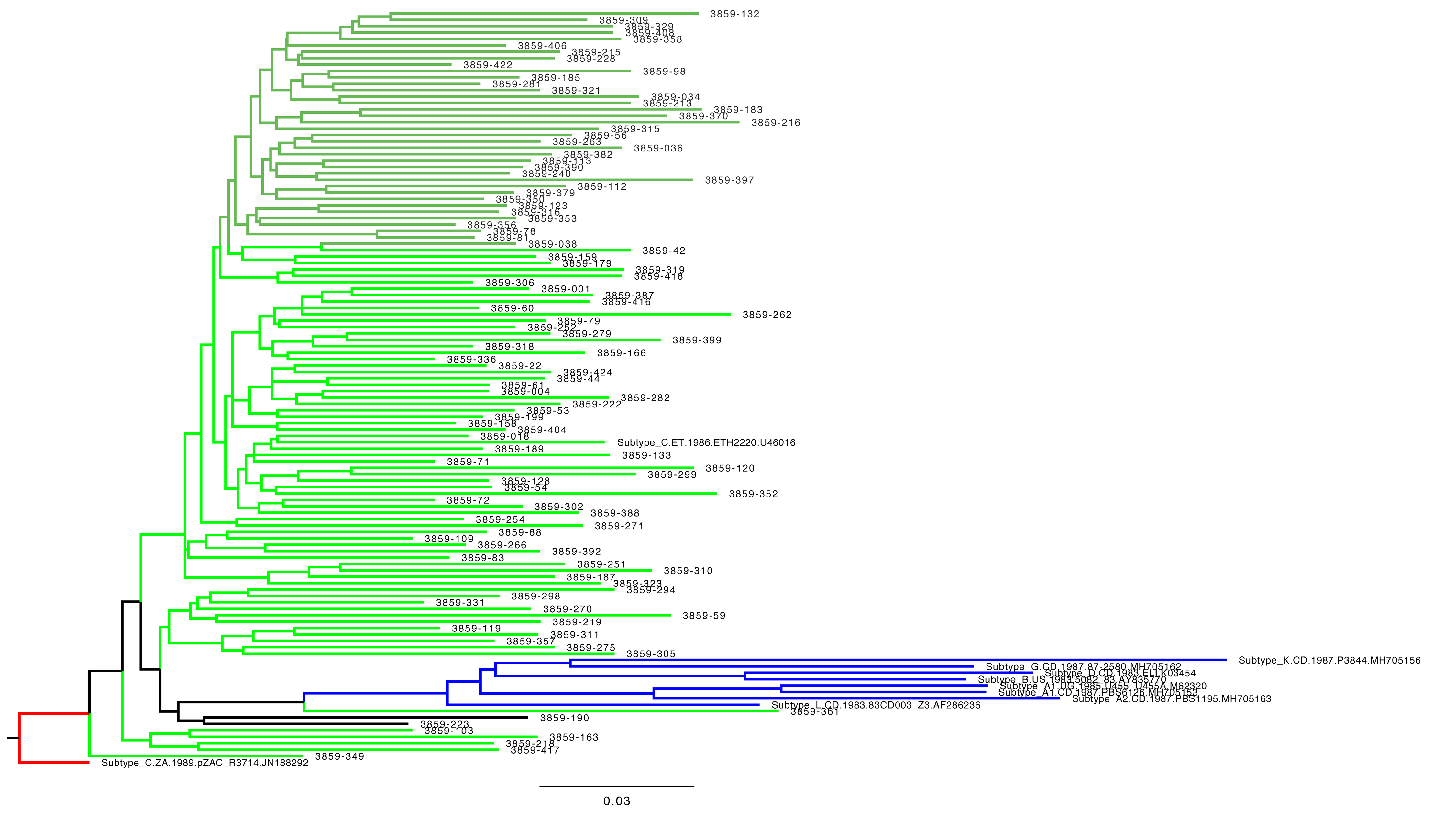

Supplement: S1 Fig — Maximum-likelihood phylogenetic tree including all sequences from participants in this study together with HIV-1 reference sequences in a rectangular layout. The tree was inferred using the generalized time reversible model with a proportion of invariant sites and gamma-distributed rate variation among sites (GTR + I + G). Branch support was assessed with 1,000 bootstrap replicates. Branch lengths represent the number of nucleotide substitutions per site (scale bar = 0.03). (TIF) [file ppat.1014118.s001.tif]

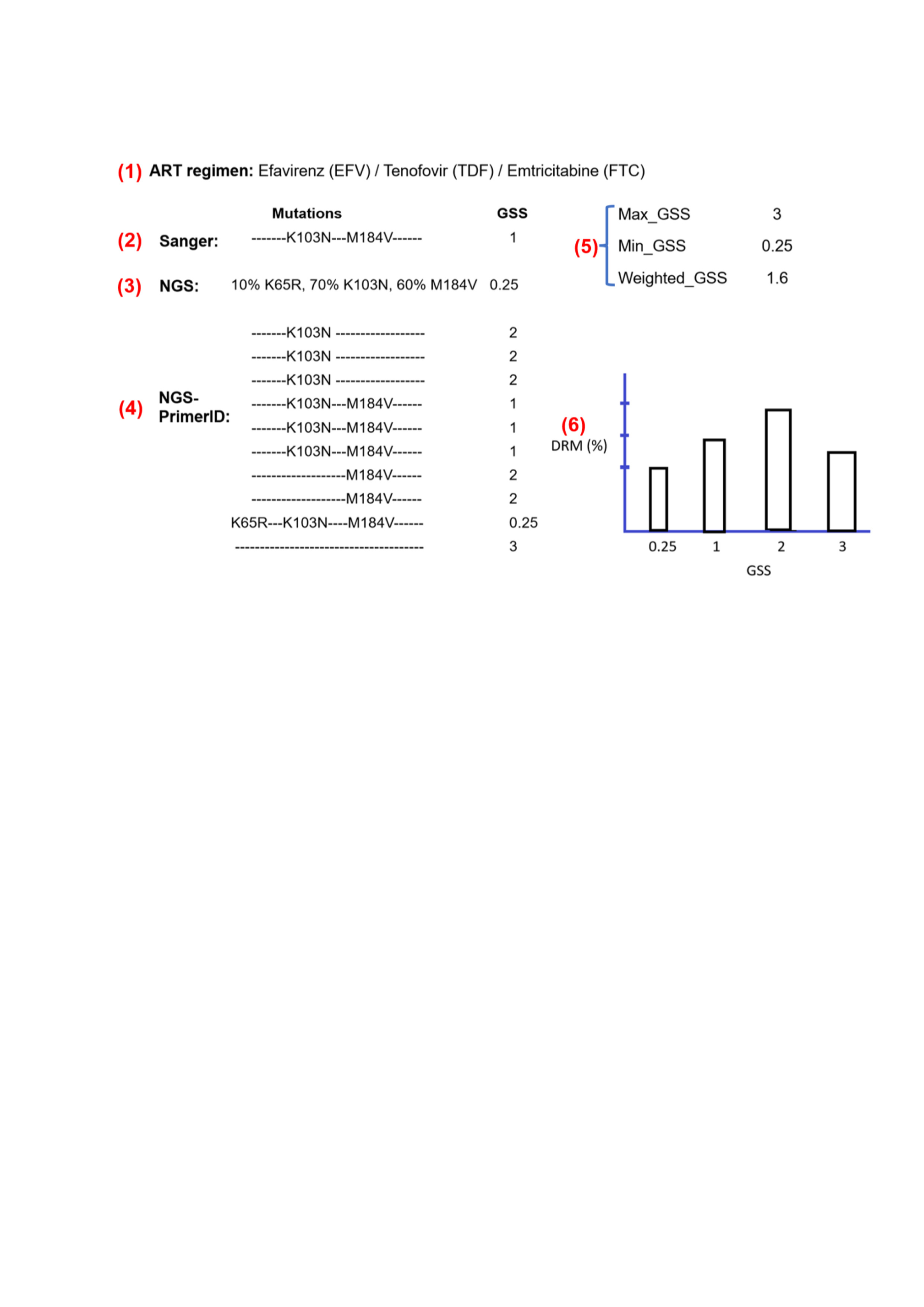

Supplement: S2 Fig — The figure shows a hypothetical participant receiving an antiretroviral regimen consisting of efavirenz (EFV), tenofovir (TDF), and emtricitabine (FTC) (1). Example outputs are shown for Sanger sequencing (2), next-generation sequencing (NGS) (3), and NGS-based ultrasensitive single-genome sequencing with primer identifiers (NGS-PrimerID) (4). For each approach, detected drug resistance mutations are used to derive regimen-level GSS values. The schematic also illustrates summary GSS metrics derived from variant-level data, including maximum GSS, minimum GSS, and weighted GSS (5), and a conceptual distribution of GSS values across detected variants (6). (TIF) [file ppat.1014118.s002.tif]

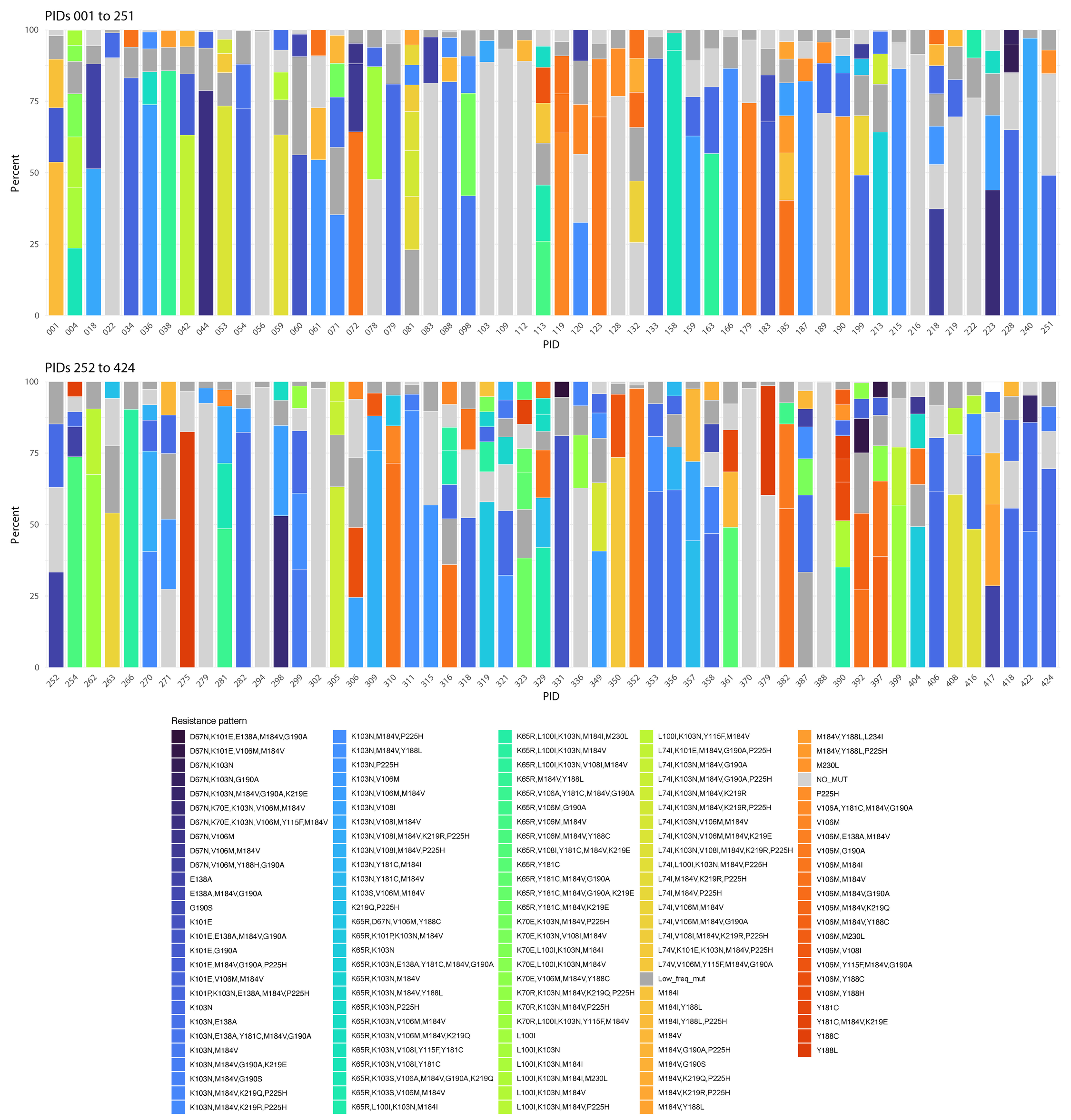

Supplement: S3 Fig — Each stacked bar plot corresponds to an individual participant, and the colors represent specific mutational patterns. Pattern frequencies were calculated from the number of sequences assigned to each pattern relative to the total number of input sequences in the sample. Reported DRM patterns present at frequencies ≤5% are shown in dark grey. (TIF) [file ppat.1014118.s003.tif]

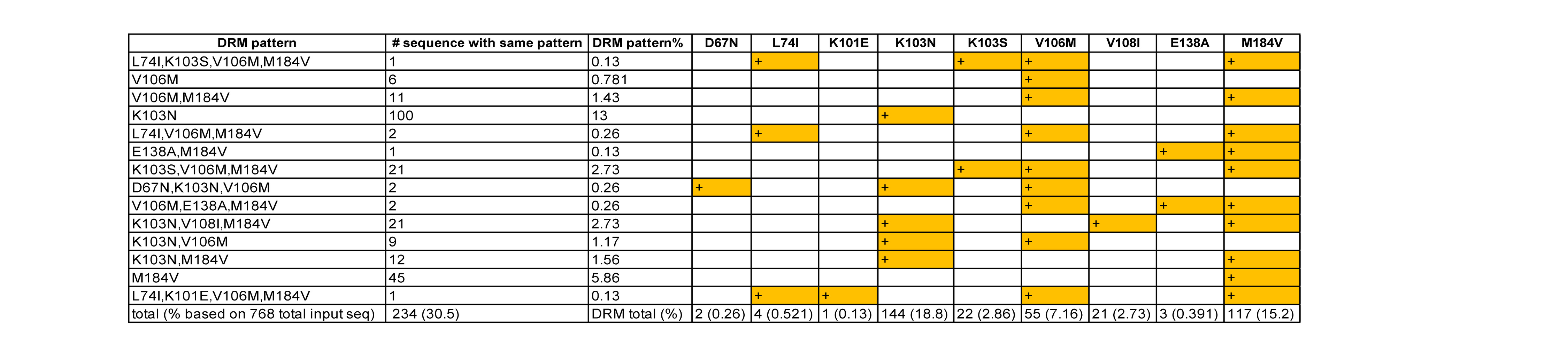

Supplement: S4 Fig — This figure illustrates a specific case where conventional Sanger sequencing underestimated the complexity of the reported resistance-pattern landscape in the sample. The first column identifies distinct mutational patterns, clarifying whether resistance markers are physically linked on individual viral genomes. The second and third columns quantify these patterns by listing the total number of sequences identified and their corresponding percentages within the specimen. Subsequent columns denote the presence of specific drug resistance mutations, which are highlighted with orange boxes. The final row provides the cumulative frequency of each individual mutation relative to the total number of input sequences in the sample. (TIF) [file ppat.1014118.s004.tif]

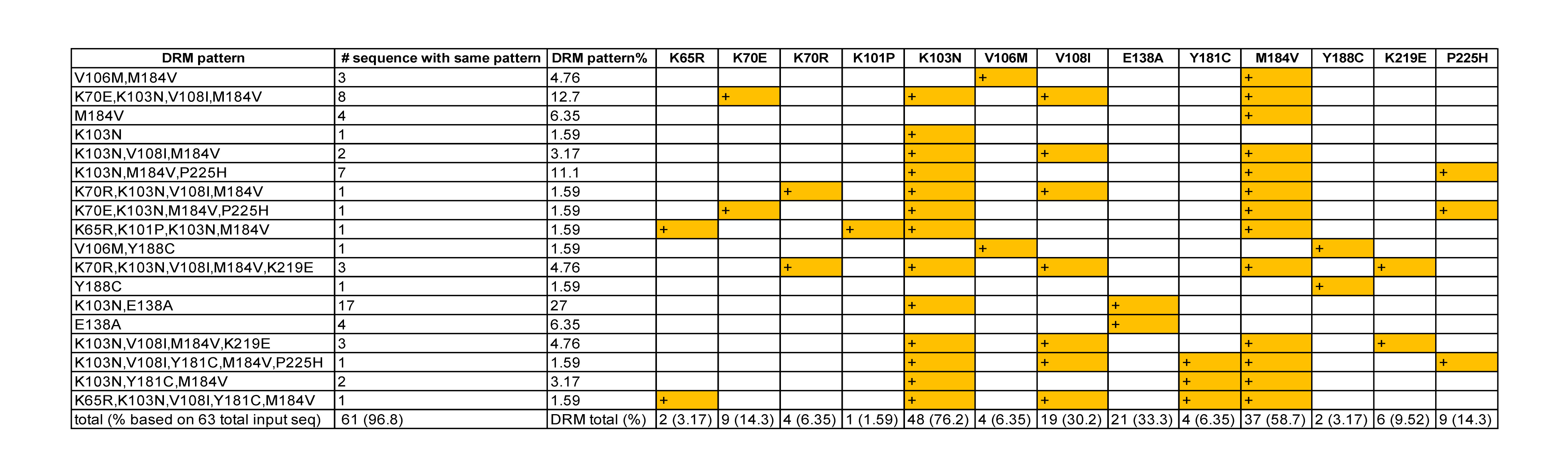

Supplement: S5 Fig — This figure illustrates a specific case where conventional Sanger sequencing overestimated the complexity of the reported resistance-pattern landscape in the sample. The first column identifies distinct mutational patterns, clarifying whether resistance markers are physically linked on individual viral genomes. The second and third columns quantify these patterns by listing the total number of sequences identified and their corresponding percentages within the specimen. Subsequent columns denote the presence of specific drug resistance mutations, which are highlighted with orange boxes. The final row provides the cumulative frequency of each individual mutation relative to the total number of input sequences in the sample. (TIF) [file ppat.1014118.s005.tif]
