# Supplementary material for: Extensive diversity and impact of drug-resistant HIV-1 variants in individuals with prior virologic failure
Source: PLoS Pathog. 2026 May 12;22(5):e1014118. doi: 10.1371/journal.ppat.1014118 (PMC13221146; doi:10.1371/journal.ppat.1014118)
Supplement: S1 Table — (DOCX) [file ppat.1014118.s006.docx]

**S1 Table: Sample-level clinical, Sanger genotypic susceptibility, and NGS-Primer ID pattern summary metrics at HIV viral load time point 1.**

| **Patient_ID** | **Sex(M/F)** | **Age** | **CD4_**  **Baseline** | **Regimens** | **HIVVL Baseline** | **NRTI (SS)** | **NNRTI (SS)** | **FTC_GSS_score** | **TDF_GSS_score** | **EFV_GSS_score** | **Sanger_Gross_GSS_Score** | **Total number of patterns identified, n** | **Total number of sequences across all patterns, n** |
| --- | --- | --- | --- | --- | --- | --- | --- | --- | --- | --- | --- | --- | --- |
| 1 | F | 37 | 354 | EFV, FTC, TDF | 175388 |  |  | 1 | 1 | 0 | 3 | 16 | 2153 |
| 22 | M | 29 | 36 | EFV, FTC, TDF | 6537 |  |  | 1 | 1 | 0 | 3 | 3 | 92 |
| 56 | M | 36 | 35 | EFV, FTC, TDF | 612883 |  |  | 1 | 1 | 0 | 3 | 12 | 8956 |
| 78 | M | 30 | 345 | EFV, FTC, TDF | 107470 |  |  | 1 | 1 | 0 | 3 | 9 | 651 |
| 83 | F | 33 | 159 | EFV, FTC, TDF | 226000 |  | E138A | 1 | 1 | 1 | 3 | 7 | 553 |
| 109 | M | 18 | 541 | EFV, FTC, TDF | 4459 |  |  | 1 | 1 | 0 | 3 | 4 | 283 |
| 183 | F | 25 | 507 | EFV, FTC, TDF | 4730 |  |  | 1 | 1 | 0 | 3 | 7 | 183 |
| 187 | F | 42 | 1275 | EFV, FTC, TDF | 108000 |  |  | 1 | 1 | 0 | 3 | 5 | 50 |
| 279 | M | 31 | 273 | EFV, FTC, TDF | 583558 |  |  | 1 | 1 | 0 | 3 | 15 | 3601 |
| 370 | F | 40 | 611 | EFV, FTC, TDF | 7192 |  |  | 1 | 1 | 0 | 3 | 4 | 508 |
| 379 | F | 22 | 753 | EFV, FTC, TDF | 23134 |  |  | 1 | 1 | 0 | 3 | 10 | 1069 |
| 18 | F | 28 | 101 | EFV, FTC, TDF | 212220 | M184V | K103N,V108I | 0 | 1 | 0 | 1 | 10 | 502 |
| 34 | F | 56 | 74 | EFV, FTC, TDF | 64138 | M184V | K101P,K103N,E138A,P225H | 0 | 1 | 0 | 1 | 17 | 297 |
| 36 | M | 60 | 21 | EFV, FTC, TDF | 69182 | M184MV | K103KN,V106VM | 0 | 1 | 0 | 1 | 14 | 374 |
| 42 | F | 36 | 398 | EFV, FTC, TDF | 12681 | L74I,M184V | K101E,G190A,P225H | 0 | 1 | 0 | 1 | 19 | 608 |
| 53 | M | 40 | 331 | EFV, FTC, TDF | 2074 | M41L,L74I,M184V,K219KDEN | K103N,V106M | 0 | 1 | 0 | 1 | 10 | 60 |
| 60 | F | 29 | 492 | EFV, FTC, TDF | 6819 | D67G | V106M,Y188YC | 1 | 1 | 0 | 2 | 17 | 64 |
| 61 | F | 18 | 331 | EFV, FTC, TDF | 2445 |  | K103N,P225H | 1 | 1 | 0 | 2 | 4 | 22 |
| 72 | M | 39 | 476 | EFV, FTC, TDF | 4544 | D67DN,M184V | V106M,F227L | 0 | 1 | 0 | 1 | 5 | 42 |
| 79 | M | 53 | 251 | EFV, FTC, TDF | 1558 |  | K103N | 1 | 1 | 0 | 2 | 5 | 21 |
| 88 | M | 45 | 624 | EFV, FTC, TDF | 69998 | M184V | K103N | 0 | 1 | 0 | 1 | 7 | 517 |
| 98 | M | 25 | 355 | EFV, FTC, TDF | 40470 | L74LI,M184V | A98G,K103N,E138G,P225H | 0 | 1 | 0 | 1 | 11 | 739 |
| 103 | M | 43 | 219 | EFV, FTC, TDF | 271201 |  |  | 1 | 1 | 0 | 3 | 4 | 53 |
| 112 | M | 44 | 139 | EFV, FTC, TDF | 1834 |  |  | 1 | 1 | 0 | 3 | 5 | 82 |
| 113 | F | 39 | 235 | EFV, FTC, TDF | 9807 |  |  | 1 | 1 | 0 | 3 | 25 | 799 |
| 119 | M | 50 | 113 | EFV, FTC, TDF | 2177293 | M184MV | V106M,V179VD | 0 | 1 | 0 | 1 | 24 | 4837 |
| 123 | F | 24 | 417 | EFV, FTC, TDF | 228387 |  | V106M,G190GA | 1 | 1 | 0 | 2 | 29 | 7303 |
| 128 | M | 51 | 281 | EFV, FTC, TDF | 33332 |  |  | 1 | 1 | 0 | 3 | 8 | 185 |
| 132 | F | 36 | 433 | EFV, FTC, TDF | 12313 | L74LI,M184V | V106M,G190A | 0 | 1 | 0 | 1 | 19 | 219 |
| 133 | M | 34 | 66 | EFV, FTC, TDF | 37493 |  | K103N | 1 | 1 | 0 | 2 | 17 | 1610 |
| 159 | F | 44 | 225 | EFV, FTC, TDF | 799000 |  | K103N,V106M | 1 | 1 | 0 | 2 | 33 | 3704 |
| 166 | F | 33 | 234 | EFV, FTC, TDF | 3294 | M184V | K103N,P225H | 0 | 1 | 0 | 1 | 13 | 303 |
| 179 | M | 38 | 248 | EFV, FTC, TDF | 73400 | M184V | V106M,E138A,V179D | 0 | 1 | 0 | 1 | 11 | 1487 |
| 185 | M | 34 | 395 | EFV, FTC, TDF | 12200 | M184V | K103KN,V106M,V179VD | 0 | 1 | 0 | 1 | 20 | 409 |
| 189 | M | 32 | 698 | EFV, FTC, TDF | 51000 |  |  | 1 | 1 | 0 | 3 | 7 | 460 |
| 190 | M | 49 | 200 | EFV, FTC, TDF | 1620 | T69D,M184V | V179D,Y188L | 0 | 1 | 0 | 1 | 5 | 33 |
| 199 | F | 34 | 261 | EFV, FTC, TDF | 22600 |  | K103N | 1 | 1 | 0 | 2 | 21 | 523 |
| 215 | M | 42 | 538 | EFV, FTC, TDF | 2690 | M184V | K103N,P225H | 0 | 1 | 0 | 1 | 3 | 22 |
| 216 | F | 45 | 319 | EFV, FTC, TDF | 47100 |  |  | 1 | 1 | 0 | 3 | 13 | 397 |
| 218 | F | 32 | 1200 | EFV, FTC, TDF | 11100 | D67N | V106M, Y188H, G190A | 1 | 1 | 0 | 2 | 21 | 335 |
| 219 | M | 56 | 213 | EFV, FTC, TDF | 123000 |  |  | 1 | 1 | 0 | 3 | 15 | 768 |
| 228 | F | 28 | 525 | EFV, FTC, TDF | 48000 | D67N | K103N,G190A | 1 | 1 | 0 | 2 | 4 | 20 |
| 240 | F | 45 | 423 | EFV, FTC, TDF | 1020 | M184V | K103N,V108I | 0 | 1 | 0 | 1 | 3 | 34 |
| 251 | F | 33 | 307 | EFV, FTC, TDF | 14300 |  | K103N | 1 | 1 | 0 | 2 | 6 | 267 |
| 254 | F | 34 | 93 | EFV, FTC, TDF | 2420 | NONE | V179D | 1 | 1 | 0.75 | 2.75 | 5 | 19 |
| 263 | F | 33 | 269 | EFV, FTC, TDF | 8980 | L74I,M184V | V106M,H221Y,F227L | 0 | 1 | 0 | 1 | 19 | 374 |
| 271 | M | 54 | 98 | EFV, FTC, TDF | 38424 |  | K103N,P225PH | 1 | 1 | 0 | 2 | 19 | 274 |
| 275 | M | 39 | 81 | EFV, FTC, TDF | 55871 |  | Y188L | 1 | 1 | 0 | 2 | 12 | 811 |
| 282 | F | 23 | 4 | EFV, FTC, TDF | 25414 |  | K103N,V106VM | 1 | 1 | 0 | 2 | 11 | 1155 |
| 294 | M | 41 | 61 | EFV, FTC, TDF | 87843 |  |  | 1 | 1 | 0 | 3 | 22 | 3924 |
| 302 | M | 52 | 613 | EFV, FTC, TDF | 1695 |  |  | 1 | 1 | 0 | 3 | 2 | 42 |
| 306 | F | 30 | 701 | EFV, FTC, TDF | 3856 |  | K103KN,V106M,Y188C | 1 | 1 | 0 | 2 | 12 | 49 |
| 309 | F | 24 | 366 | EFV, FTC, TDF | 1270 |  | K103KN,V108VI | 1 | 1 | 0 | 2 | 5 | 25 |
| 310 | M | 40 | 747 | EFV, FTC, TDF | 5103 | M184V | V106M,F227L | 0 | 1 | 0 | 1 | 6 | 84 |
| 311 | M | 31 | 364 | EFV, FTC, TDF | 2508 | M184V | K103N,P225H | 0 | 1 | 0 | 1 | 6 | 90 |
| 315 | F | 44 | 154 | EFV, FTC, TDF | 10117 |  | K103N,P225PH | 1 | 1 | 0 | 2 | 12 | 537 |
| 316 | F | 18 | 313 | EFV, FTC, TDF | 3403 | M184V | K103N,P225H | 0 | 1 | 0 | 1 | 10 | 25 |
| 331 | M | 30 | 563 | EFV, FTC, TDF | 5577 | M184V | E138A | 0 | 1 | 1 | 2 | 17 | 201 |
| 336 | F | 31 | 413 | EFV, FTC, TDF | 25787 | M41L,K70R,M184V,K219Q | K103N,P225H | 0 | 1 | 0 | 1 | 31 | 1234 |
| 349 | M | 36 | 56 | EFV, FTC, TDF | 43022 | L74LI,M184V | K103N,V106M | 0 | 1 | 0 | 1 | 23 | 848 |
| 350 | F | 50 | 30 | EFV, FTC, TDF | 250976 | L74V,Y115F,M184V | V106M,V179D,G190A | 0 | 1 | 0 | 1 | 21 | 1825 |
| 352 | F | 53 | 441 | EFV, FTC, TDF | 2261 | M184V | V106M,V179D | 0 | 1 | 0 | 1 | 3 | 84 |
| 356 | M | 54 | 547 | EFV, FTC, TDF | 1720 | M184V | K103N | 0 | 1 | 0 | 1 | 13 | 140 |
| 357 | M | 49 | 44 | EFV, FTC, TDF | 30714 | M184V | K103NS,V106VM,V108VI | 0 | 1 | 0 | 1 | 7 | 397 |
| 358 | F | 31 | 24 | EFV, FTC, TDF | 681271 |  | K103N | 1 | 1 | 0 | 2 | 22 | 1492 |
| 361 | M | 44 | 332 | EFV, FTC, TDF | 7219 | M184V | K103N,V108I,P225H | 0 | 1 | 0 | 1 | 17 | 1209 |
| 382 | M | 25 | 494 | EFV, FTC, TDF | 32200 |  | V106M,G190A | 1 | 1 | 0 | 2 | 6 | 27 |
| 388 | M | 53 | 477 | EFV, FTC, TDF | 53274 |  |  | 1 | 1 | 0 | 3 | 3 | 1057 |
| 390 | M | 43 | 57 | EFV, FTC, TDF | 35177 |  | V106M,Y188C | 1 | 1 | 0 | 2 | 10 | 37 |
| 392 | F | 39 | 44 | EFV, FTC, TDF | 5389 | D67DGNS,M184V | K101KE,V106M,Y188YC,F227L | 0 | 1 | 0 | 1 | 32 | 449 |
| 397 | M | 23 | 604 | EFV, FTC, TDF | 2898 | M184V, D67N | K101P,K103N,E138A | 0 | 1 | 0 | 1 | 31 | 914 |
| 399 | F | 41 | 502 | EFV, FTC, TDF | 6088 |  |  | 1 | 1 | 0 | 3 | 18 | 523 |
| 404 | F | 31 | 58 | EFV, FTC, TDF | 29574 |  | K103N | 1 | 1 | 0 | 2 | 14 | 150 |
| 406 | F | 50 | 289 | EFV, FTC, TDF | 43700 |  | V106M,Y188H | 1 | 1 | 0 | 2 | 9 | 107 |
| 408 | F | 40 | 351 | EFV, FTC, TDF | 20351 |  | K103N | 1 | 1 | 0 | 2 | 9 | 119 |
| 417 | F | 21 | 388 | EFV, FTC, TDF | 67834 |  | K103N | 1 | 1 | 0 | 2 | 6 | 28 |
| 418 | F | 64 | 771 | EFV, FTC, TDF | 1140 |  | K103N | 1 | 1 | 0 | 2 | 8 | 97 |
| 422 | M | 36 | 123 | EFV, FTC, TDF | 1292 | D67D/N,M184V,K219E | K103N, G109A | 0 | 1 | 0 | 1 | 4 | 21 |
| 424 | F | 29 | 347 | EFV, FTC, TDF | 69737 |  | K103N | 1 | 1 | 0 | 2 | 5 | 23 |
| 353 | M | 26 | 1234 | EFV, FTC, TDF | 9440 | M184V | K103N,P225H | 0 | 1 | 0 | 1 | 6 | 26 |
| 416 | M | 28 | 23 | EFV, FTC, TDF | 205571 | M184V,L74I | L100I,K103N,P225H | 0 | 1 | 0 | 1 | 8 | 62 |
| 299 | M | 46 | 219 | EFV, FTC, TDF | 1043 | M184V | K103N, P225H | 0 | 1 | 0 | 1 | 6 | 64 |
| 318 | F | 41 | 204 | EFV, FTC, TDF | 10038 | D67N,M184V,K219E | V106A | 0 | 1 | 0.25 | 1.25 | 5 | 21 |
| 4 | M | 31 | 280 | EFV, FTC, TDF | 157415 | D67G,K70KE,M184V | L100I,K103N | 0 | 0.5 | 0 | 0.5 | 24 | 550 |
| 38 | F | 49 | 379 | EFV, FTC, TDF | 23787 | K65R,M184V | Y188L | 0 | 0.25 | 0 | 0.25 | 17 | 745 |
| 44 | M | 33 | 98 | EFV, FTC, TDF | 18635 | D67N,K70E,Y115F,M184V | K103N,V106M | 0 | 0.25 | 0 | 0.25 | 27 | 625 |
| 222 | F | 46 | 146 | EFV, FTC, TDF | 1450 | A62AV,K65R,M184V | L100I,K103N | 0 | 0.25 | 0 | 0.25 | 14 | 193 |
| 223 | F | 30 | 2 | EFV, FTC, TDF | 634000 | M184V | Y188L | 0 | 1 | 0 | 1 | 18 | 385 |
| 213 | M | 47 | 55 | EFV, FTC, TDF | 305000 | K65R,M184V | K101H,K103N,E138A,Y181C,G190A,H221Y | 0 | 0.25 | 0 | 0.25 | 36 | 886 |
| 262 | F | 31 | 28 | EFV, FTC, TDF | 198633 | K65N,K70R,Y115F,M184V | L100I,K103N,H221Y | 0 | 0.25 | 0 | 0.25 | 27 | 3048 |
| 266 | M | 42 | 16 | EFV, FTC, TDF | 999 | A62AV,K65R,M184V | L100I,K103N | 0 | 0.25 | 0 | 0.25 | 18 | 1343 |
| 270 | M | 38 | 726 | EFV, FTC, TDF | 4325 | K70N,M184V,K219R | K103N,V108I,P225H | 0 | 0.5 | 0 | 0.5 | 6 | 37 |
| 281 | F | 30 | 328 | EFV, FTC, TDF | 2459 | K65R,M184V,K219Q | K103GS,V106A,G190A | 0 | 0.25 | 0 | 0.25 | 6 | 35 |
| 298 | M | 41 | 457 | EFV, FTC, TDF | 8823 | A62AV,K65KR,D67DN,K70KE,L74LI,M184V | K103N,V106M | 0 | 0 | 0 | 0 | 16 | 671 |
| 305 | M | 29 | 290 | EFV, FTC, TDF | 20469 | K70Q,L74I,M184V,K219KR | A98AG,K103N,G190GA,F227L | 0 | 0.5 | 0 | 0.5 | 20 | 204 |
| 323 | F | 41 | 280 | EFV, FTC, TDF | 14087 | K65R,M184V,K219E | Y181C,G190GA | 0 | 0.25 | 0 | 0.25 | 14 | 47 |
| 329 | M | 39 | 278 | EFV, FTC, TDF | 14453 | A62V,K65R,K70KT,M184V | K103NS,V106M | 0 | 0 | 0 | 0 | 12 | 138 |
| 71 | F | 41 | 343 | EFV, FTC, TDF | 8980 | K70KE,M184V | K103N,P225H | 0 | 0.5 | 0 | 0.5 | 16 | 51 |
| 158 | F | 58 | 386 | EFV, FTC, TDF | 5800 | K65R,Y115YF | K103N,V108I,Y181C,H221HY | 0.5 | 0 | 0 | 0.5 | 7 | 620 |
| 163 | F | 42 | 257 | EFV, FTC, TDF | 2280 | K65R,M184V | A98G,L100I,K103N,V108I | 0 | 0.25 | 0 | 0.25 | 5 | 30 |
| 54 | F | 25 | 8 | EFV, FTC, TDF | 161582 | K65R,T69Deletion | V106M,Y181C,H221Y | 0.25 | 0 | 0 | 0.25 | 14 | 315 |
| 59 | M | 32 | 629 | EFV, FTC, TDF | 6931 | K65R,Y115F | K103N,V106M | 0.5 | 0 | 0 | 0.5 | 16 | 155 |
| 81 | M | 39 | 286 | EFV, FTC, TDF | 3696 | K70KN,L74I,Y115YF,M184V,K219R | A98G,K103N,V108VI,P225H | 0 | 0.25 | 0 | 0.25 | 38 | 1072 |
| 120 | F | 41 | 223 | EFV, FTC, TDF | 40389 | K65R,T69del | V106M,Y181C,Y188C | 0.25 | 0 | 0 | 0.25 | 9 | 46 |
| 252 | F | 48 | 55 | EFV, FTC, TDF | 2199 | K65R,M184V,K219E | K103N,V108I,M230L,L234I | 0 | 0.25 | 0 | 0.25 | 7 | 27 |
| 319 | F | 34 | 235 | EFV, FTC, TDF | 1386 | A62AV,K65R,M184V | K101P,K103N,V179L | 0 | 0.25 | 0 | 0.25 | 7 | 19 |
| 321 | M | 34 | 112 | EFV, FTC, TDF | 2483 | K65R,M184V | K103N,Y188L | 0 | 0.25 | 0 | 0.25 | 8 | 31 |
| 387 | F | 26 | 87 | EFV, FTC, TDF | 3139 | D67DN,K70KEGR,M184V,T215I,K219E | K103N,V108I | 0 | 0.25 | 0 | 0.25 | 19 | 63 |

**PID, participant identifier; HIVVL, HIV viral load; mL; millilitre; mm^3^, cubic millimetres; GSS, genotypic susceptibility scores; Efavirenz, EFV); Emtricitabine, FTC; Tenofovir, TDF; CD4, cluster of differentiation 4; NRTI, Nucleoside/Nucleotide Reverse Transcriptase Inhibitor; NNRTI, non-nucleoside reverse transcriptase inhibitor. Pattern counts include both DRM-containing patterns and the pattern representing sequences without detected DRMs.**
