# Supplementary material for: Extensive diversity and impact of drug-resistant HIV-1 variants in individuals with prior virologic failure
Source: PLoS Pathog. 2026 May 12;22(5):e1014118. doi: 10.1371/journal.ppat.1014118 (PMC13221146; doi:10.1371/journal.ppat.1014118)
Supplement: S2 Table — (DOCX) [file ppat.1014118.s007.docx]

**S2 Table: Genotypic Susceptibility Scores with corresponding susceptibility levels**

| **Total Penalty Score** | **Susceptibility Level** | **Genotypic Susceptibility**  **Scores** |
| --- | --- | --- |
| <10 | Susceptible | 1 |
| 10 to <15 | Potential low-level resistance | 0.75 |
| 15 to <30 | Low-level resistance | 0.5 |
| 30 to <60 | Intermediate resistance | 0.25 |
| ≥60 | High-level resistance | 0 |
