# Supplementary material for: Extensive diversity and impact of drug-resistant HIV-1 variants in individuals with prior virologic failure
Source: PLoS Pathog. 2026 May 12;22(5):e1014118. doi: 10.1371/journal.ppat.1014118 (PMC13221146; doi:10.1371/journal.ppat.1014118)
Supplement: S3 Table — (DOCX) [file ppat.1014118.s008.docx]

**S3 Table: Selection Criteria for Plasma specimens GSS ≥1**

| **Selection Criteria** | **SOC** | **RT** | **Total** |
| --- | --- | --- | --- |
| Patient sequences (TP 1) | 210 | 170 | 380 |
| Patient sequences with GSS ≥1(TP 1) | 87 | 83 | 170 |
| Patients with GSS ≥1 (TP 1), maintained on first-line ART with VF at (TP 3) (HIVVL ≥1000 cp/ml) | 62 | 5 | 67 |
| Patients with GSS ≥1, maintained on first-line ART, without VF at Time Point 3 (HIVVL<1000 cp/ml) | 20 | 6 | 26 |

**VF = Virologic Failure, GSS = Genotypic Susceptibility Scores; SOC = Standard of Care. RT = Resistance Testing; VL = Viral load; ART=Antiretroviral therapy; TP = Timepoint**
