# Supplementary material for: Extensive diversity and impact of drug-resistant HIV-1 variants in individuals with prior virologic failure
Source: PLoS Pathog. 2026 May 12;22(5):e1014118. doi: 10.1371/journal.ppat.1014118 (PMC13221146; doi:10.1371/journal.ppat.1014118)
Supplement: S4 Table — (DOCX) [file ppat.1014118.s009.docx]

**S4 Table: Selection criteria used for Plasma specimens with GSS <1**

| **Selection Criteria** | **SOC** |
| --- | --- |
| Patient sequences (TP 1) | 210 |
| Patient sequences with GSS <1 (TP 1) | 101 |
| Patients with GSS <1 (TP 1) without VF at TP 2  (HIVVL <1000 cp/ml) | 14 |
| Patients with GSS <1 with VF at TP 2 (HIVVL≥1000 cp/ml). | 14 |

**VF = Virologic Failure; GSS = Genotypic Susceptibility Scores; SOC = Standard of Care;**

**Viral load = VL; ART = Antiretroviral therapy; TP = Timepoint**
