# Supplementary material for: Extensive diversity and impact of drug-resistant HIV-1 variants in individuals with prior virologic failure
Source: PLoS Pathog. 2026 May 12;22(5):e1014118. doi: 10.1371/journal.ppat.1014118 (PMC13221146; doi:10.1371/journal.ppat.1014118)
Supplement: S5 Table — (DOCX) [file ppat.1014118.s010.docx]

**S5 Table: Participant specimens selected from the REVAMP Study for NGS-Primer ID**

| **Study Arm** | **Number of specimens** | **Time Point** | **Sanger-based GSS** | **ART Maintained or Switched** | **Assay** | **Analysis** |
| --- | --- | --- | --- | --- | --- | --- |
| RT and SOC | 93 | 1 | ≥1 | Maintained on  first-line ART | NGS-Primer ID | NGS-derived GSS and  Resistance Linkage |
| SOC | 14 | 1 | <1 |  |  |  |

**RT, resistance testing; SOC, standard of care, ART, antiretroviral therapy, NGS-Primer ID; next generation sequencing-ultrasensitive single genome sequencing.**

**GSS, genotypic susceptibility scores**
