# Supplementary material for: Extensive diversity and impact of drug-resistant HIV-1 variants in individuals with prior virologic failure
Source: PLoS Pathog. 2026 May 12;22(5):e1014118. doi: 10.1371/journal.ppat.1014118 (PMC13221146; doi:10.1371/journal.ppat.1014118)
Supplement: S6 Table — (DOCX) [file ppat.1014118.s011.docx]

**S6 Table: cDNA Primers with Primer ID tags used for reverse transcription**

| **Primer Name** | **Primer Sequence (5’-3’)** |
| --- | --- |
| **GSPID-3271R C** | GGTATCGAAGTCATCCTGCTAGNNNNNNNNNNACTGTCCATTTGTCAGGATG |
| **GSPID-3271R C (Tvar)** | GGTATCGAAGTCATCCTGCTAGNNNNNNNNNNACTGTCCATTT**A**TCAGGATG |
| **GSPID-3271R C (Gvar)** | GGTATCGAAGTCATCCTGCTAGNNNNNNNNNNACTGTCCA**C**TTGTCAGGATG |
| **GSPID-3271R C (Cvar)** | GGTATCGAAGTCATCCTGCTAGNNNNNNNNNNACTGTCCATTTGTCAGG**G**TG |
| **GSPID-3271R C (ACvar):** | GGTATCGAAGTCATCCTGCTAGNNNNNNNNNNACTGTCCATTTG**GT**AGGATG |

Primer sequence consists of priming region (Green), 10bp Primer ID tag (Blue), gene specific region (Orange) and base pairs that are different in the variant specific primers compared to the GSPID-3271R (red bold)
