# Supplementary material for: Extensive diversity and impact of drug-resistant HIV-1 variants in individuals with prior virologic failure
Source: PLoS Pathog. 2026 May 12;22(5):e1014118. doi: 10.1371/journal.ppat.1014118 (PMC13221146; doi:10.1371/journal.ppat.1014118)
Supplement: S7 Table — (DOCX) [file ppat.1014118.s012.docx]

**S7 Table: Complementary DNA synthesis Master Mix 1**

| **Reagent** | |  | **Volume per reaction (µl)** | |
| --- | --- | --- | --- | --- |
| **Nuclease-free Water** | |  | | **0.0** |
| **Deoxynucleotide triphosphate (dNTP)** | |  | | 2.5 |
| **GSPID Primer (5µM)** | |  | | 2.5 |
| **RNA** | |  | | **20** |
| **Total Volume** | |  | | 25 |
| **Thermocycling Conditions** | | | | |
|  | **Temperature (^o^C)** | **Time** | | **Cycle(s)** |
| **Denaturation** | **85** | **10 minutes** | | **1** |
| **Cool** | **Snap-freeze** | **1 minute** | | **-** |
