# Supplementary material for: Extensive diversity and impact of drug-resistant HIV-1 variants in individuals with prior virologic failure
Source: PLoS Pathog. 2026 May 12;22(5):e1014118. doi: 10.1371/journal.ppat.1014118 (PMC13221146; doi:10.1371/journal.ppat.1014118)
Supplement: S8 Table — (DOCX) [file ppat.1014118.s013.docx]

**S8 Table: Complementary DNA synthesis Master Mix 2**

| **Reagent** | | | |  | | **Volume per reaction (µl)** | |
| --- | --- | --- | --- | --- | --- | --- | --- |
| Nuclease-free Water | | | |  | | 7.5 | |
| 5x First-strand Buffer | | | |  | | 10.0 | |
| RNase out Inh | | | |  | | 2.5 | |
| DTT (dithiothreitol) | | | |  | | 2.5 | |
| SSIV/ Platinum SuperFi DNA polymerase (2X) | | | |  | | 2.5 | |
| **Total volume** | | | |  | | **25** | |
| **Thermocycling Conditions** | | | | | | | |
|  | **Temperature (^o^C)** | | **Time** | | | | **Cycle(s)** |
|  |  |  |  | |  | |  |
| Denaturation  cDNA Synthesis |  | 85 |  | | 10 minutes | | 1 |
|  |  | **45** |  | | 1 Hour | | 1 |
| Hold |  | 4 |  | | ∞ | | Hold |
