# Supplementary material for: Extensive diversity and impact of drug-resistant HIV-1 variants in individuals with prior virologic failure
Source: PLoS Pathog. 2026 May 12;22(5):e1014118. doi: 10.1371/journal.ppat.1014118 (PMC13221146; doi:10.1371/journal.ppat.1014118)
Supplement: S10 Table — (DOCX) [file ppat.1014118.s015.docx]

**S10 Table: Second-Round Master Mix and Conditions**

| **Reagent** | | **Volume per Reaction (µl)** | |  |
| --- | --- | --- | --- | --- |
| KAPA HiFi Uracil + (2X) | | 12.5 | |  |
| Nuclease-free Water | | 6 | |  |
| 2709 forward (10µM) | | 0.75 | |  |
| primRegion-R-5Us (10µM) | | 0.75 | |  |
| **Total Volume** | | **20** | |  |
| Template | | **5µl** | |  |
| **Thermocycling Conditions** | | | | |
|  | **Temperature (^o^C)** | **Time** | **Cycle(s)** | |
| Pre-denaturation | 95 | 3 minutes | 1 | |
| Denaturation | 98 | 15 seconds | 28 | |
| Annealing | 52 | 30 seconds |  |  |
| Extension | 72 | 30 seconds |  |  |
| Final Extension | 72 | 1 minute | 1 | |
| Hold | 4 | ∞ | Hold | |
