# Supplementary material for: Extensive diversity and impact of drug-resistant HIV-1 variants in individuals with prior virologic failure
Source: PLoS Pathog. 2026 May 12;22(5):e1014118. doi: 10.1371/journal.ppat.1014118 (PMC13221146; doi:10.1371/journal.ppat.1014118)
Supplement: S11 Table — (DOCX) [file ppat.1014118.s016.docx]

**S11 Table: cDNA Amplification Primers used in First and Second Round PCR**

| **Primer Name** | **Direction** | **Primer Sequence (5’-3’)** |
| --- | --- | --- |
| 2589 FC | Forward | 5’CCAGGAATGGATGGCCCAA3’ |
| 2709 FC | Forward | 5’AAYCCAdUAdUAAYACdUCCARdUATTTG 3’ |
| PrimRegion-R-5Us | Reverse | 5′ GGdUAdUCGAAGdUCAdUCCdUGCTAG 3′ |
