# Supplementary material for: Extensive diversity and impact of drug-resistant HIV-1 variants in individuals with prior virologic failure
Source: PLoS Pathog. 2026 May 12;22(5):e1014118. doi: 10.1371/journal.ppat.1014118 (PMC13221146; doi:10.1371/journal.ppat.1014118)
Supplement: S1 Appendix — Supplementary Methods: REVAMP Study: Participant enrolment and Randomization. REVAMP Study: Inclusion and Exclusion Criteria. Plasma Specimen Selection. Next-generation sequencing with Primer ID (NGS-Primer ID). HIV drug-resistance analysis pipeline (hivdrm). Phylogenetic Analysis. Supplementary Results. Diverse range of mutational patterns observed by NGS-Primer ID across all participants. Characterization of Resistant and Susceptible variants using weighted GSS by NGS-Primer ID. (DOCX) [file ppat.1014118.s017.docx]

**Supplementary Appendix**

**Extensive diversity and impact of drug-resistant HIV-1 variants in individuals with prior virologic failure**

Table of Contents

[Supplementary Methods 3](#_Toc225415657)

[REVAMP Study: Participant enrolment and Randomization 3](#_Toc225415658)

[REVAMP Study: Inclusion and Exclusion Criteria 3](#_Toc225415659)

[Plasma Specimen Selection 4](#_Toc225415660)

[Next-generation sequencing with Primer ID (NGS-Primer ID) 4](#_Toc225415661)

[HIV drug-resistance analysis pipeline (hivdrm) 5](#_Toc225415662)

[Phylogenetic Analysis 7](#_Toc225415663)

[Supplementary Results 8](#_Toc225415664)

[Diverse range of mutational patterns observed by NGS-Primer ID across all participants. 8](#_Toc225415665)

[Characterization of Resistant and Susceptible variants using weighted GSS by NGS-Primer ID 9](#_Toc225415666)

[Supplementary Figures 11](#_Toc225415667)

[S1 Fig. Maximum likelihood phylogenetic tree 11](#_Toc225415668)

[S2 Fig. Schematic illustration of genotypic susceptibility score (GSS) estimation from different sequencing approaches 12](#_Toc225415669)

[S3 Fig. Distribution of viral mutational patterns across the study cohort 13](#_Toc225415670)

[S4 Fig. Detailed mapping of resistance patterns in PID 219 14](#_Toc225415671)

[S5 Fig. Detailed mapping of resistance patterns in PID 387 15](#_Toc225415672)

[Supplementary Tables 16](#_Toc225415673)

[S1 Table: Sample-level clinical, Sanger genotypic susceptibility, and NGS-Primer ID pattern summary metrics at HIV viral load time point 1. 16](#_Toc225415674)

[S2 Table: Genotypic Susceptibility Scores with corresponding susceptibility levels 24](#_Toc225415675)

[S3 Table: Selection Criteria for Plasma specimens GSS ≥1 24](#_Toc225415676)

[S4 Table: Selection criteria used for Plasma specimens with GSS <1 25](#_Toc225415677)

[S5 Table: Participant specimens selected from the REVAMP Study for NGS-Primer ID 25](#_Toc225415678)

[S6 Table: cDNA Primers with Primer ID tags used for reverse transcription 26](#_Toc225415679)

[S7 Table: Complementary DNA synthesis Master Mix 1 26](#_Toc225415680)

[S8 Table: Complementary DNA synthesis Master Mix 2 27](#_Toc225415681)

[S9 Table: First-Round Master Mix and Conditions 28](#_Toc225415682)

[S10 Table: Second-Round Master Mix and Conditions 29](#_Toc225415683)

[S11 Table: cDNA Amplification Primers used in First and Second Round PCR 29](#_Toc225415684)

# Supplementary Methods

## REVAMP Study: Participant enrolment and Randomization

The study timepoint (TP) 1 was participants being randomized on the day of enrolment. Those participants who failed the first-line non-nucleoside reverse transcriptase inhibitors (NNRTIs), were randomized to either receive SOC (which included intensive adherence counselling and repeat HIV viral load (HIVVL) testing in 3 to 6 months) or Sanger-based resistance testing; these participants in the RT arm had their blood taken for HIV-1 drug resistance (HIV DR testing). The study TP 2 for the SOC arm had participants returning three months after enrolment (TP 2A-SOC) for repeat viral load (VL) testing. Participants returned a month later (TP 2B-SOC) for their HIV VL results. Those with a viral load ≥1 000 copies/ml were switched to a second-line protease inhibitor (PI) based regimen while those with a viral load <1 000 copies/ml continued with their first-line antiretroviral therapy (ART). The study TP 2 for the RT arm had participants returning one month after enrolment (TP 2 RT) to review the HIV DR results. Participants who did not show drug resistance continued with their first-line regimens, while those with drug resistance were switched to a second-line PI-based regimen. The study TP 3 had SOC and RT participants returning 9 months after enrolment. The SOC and RT participants had bloods taken for HIV VL testing, with reflex HIVDR testing if viral loads were ≥1 000 copies/ml. SOC participants who had a viral load ≥1 000 copies/ml were switched to second or third-line ART and their stored plasma was tested for HIV DR. If the viral load was <1 000 copies/ml, participants continued to receive their TP 2 ART. The SOC and RT arm HIV DR results were reviewed. If the test showed resistance, participants were switched from first to second line and second to third line ART appropriately. If no resistance was detected, they continued their TP 2 ART regimen.

## REVAMP Study: Inclusion and Exclusion Criteria

Participants who lived within 100 kilometres (km) of the clinic, ≥18 years of age, HIV positive, and who had a detectable viral load of ≥1 000 copies/ml within the last 3 months were considered for enrolment into the REVAMP study. Participants were on a non-nucleoside reverse transcriptase inhibitor (NNRTI)-based ART for at least 6 months, and any regimen switches were within the NNRTI and nucleos(t)ide backbone. All participants who participated in the enrolment and follow-up visit provided informed consent. Participants were excluded from the REVAMP study, if they had known HIV drug resistance, previously received a protease inhibitor (PI)-based ART or had a clinical indication to start PI-based ART immediately (1). Participants who were not intending to remain in the clinic area for the next nine months were excluded.

## Plasma Specimen Selection

Sanger-based HIV-1 drug resistance testing was done on 381 plasma specimens at time point (TP) 1 (n=210 in the SOC arm and n=171 in the RT arm) from participants in the REVAMP study and genotypic susceptibility scores (GSS) were calculated for approximately 381 time point (TP) 1 plasma specimens **(S2-S5 Tables)** (1). One hundred and seventy-one plasma specimens (171/381) with a Sanger GSS ≥ 1 (87 in the standard of care (SOC) arm and 84 in the resistance testing (RT) arm) **(S3 Table)** were screened by Sanger sequencing for NGS-Primer ID testing. Plasma specimens with virologic failure (VF) above 1000cp/ml (62 in SOC and 6 in RT) and without VF less than 1000 cp/ml (20 in SOC and 6 in RT) were selected for NGS-Primer ID testing. Plasma specimens from SOC participants with a Sanger-based GSS <1 (N=14) who were maintained on their first-line regimen were also selected bringing the number of specimens for the characterization experiments using NGS-Primer ID to 108 (**S4-S5 Table)**. Plasma specimens were excluded from testing if there were missing HIV-1 viral load data, if the Sanger sequencing data was of poor quality, or if GSS could not be determined.

## Next-generation sequencing with Primer ID (NGS-Primer ID)

We have provided a hypothetical example **(Figure S2)** to highlight the essence of our study by showing the differences in GSS obtained from three methods:

Sanger sequencing, next-generation sequencing (NGS), and NGS-Primer ID. The hypothetical example considers a patient on an ART regimen (1) consisting of Efavirenz (EFV), Tenofovir (TDF), and Emtricitabine (FTC). By Sanger Sequencing (2) we get two different mutations: K103N and M184V. Together, the GSS score =1 for this ART regimen. The only active ARV is TDF. By NGS (3) we get 10% (K65R), 70% (K103N), 60%(M184V); together the GSS is 0.25 (K65R low-frequency mutation detected conferring intermediate resistance (IR) to TDF (drug score =40) not detected by Sanger sequencing). When using NGS-Primer ID (4), we can gain insight into the actual viral population by observing GSS scores ranging across different variants. Some variants have a score of 2, some have a score of 1, and some are the wild type (WT). This demonstrates the importance of resistance linkage in accurately characterizing the spectrum of resistant variants within the quasispecies. By analyzing this data (5), we could determine the maximum (MAX), minimum (MIN), and weighted GSS (wGSS). The wGSS was calculated (6) using a specific formula: wGSS = sum of the GSS multiplied by the percentage of the drug-resistant mutations (DRMs). Weighted GSS was our primary method for assessing the impact of virologic (VF) in our study. NGS-Primer ID characterised the diversity of DRMs (majority or minority, linked or unlinked) in participants with virologic failure on first-line NNRTI-based ART.

## HIV drug-resistance analysis pipeline (hivdrm)

HIV drug-resistance analyses were performed using a modified derivative of the hivdrm workflow run on paired-end FASTQ files together with a four-column barcode file (Sample_ID, Primers, F-Linkers, R-Linkers) and a user-supplied edited HIV reference FASTA provided with the --reference argument. The workflow is reference-driven rather than coordinate-hardcoded, such that the genomic interval analyzed is defined by the reference sequence used for each run. Paired-end reads were processed by reverse-complementing read 2 and concatenating it to read 1 to reconstruct a single amplicon sequence. Reads were retained only if at least 90% of bases had Phred quality scores ≥20, converted to FASTA format, and trimmed by 4 nucleotides from both the 5′ and 3′ ends before demultiplexing. Sample assignment was then performed using the combined barcode formed from the forward linker and the reverse-complement of the reverse linker defined in the barcode file.

A BLAST nucleotide database was created from the supplied reference FASTA, and demultiplexed reads were aligned using blastn-short with word_size=4, dust=no, soft_masking=false, num_alignments=5, and XML output. UMI extraction was performed by identifying a run of at least 5 consecutive N bases in the aligned reference sequence and taking the corresponding aligned query segment as the raw UMI. UMIs differing by a Hamming distance of 1 could be collapsed into the same family. For each UMI family, insertions in the query relative to the reference were removed before consensus generation. Consensus sequences were then generated only for UMI families with at least 5 reads and identical family-member lengths, using an 80% nucleotide agreement threshold at each position; positions not meeting this threshold were assigned as N.

Drug-resistance interpretation was performed using SierraPy/Stanford HIVdb (2). Because newer sierrapy versions can produce chunked JSON outputs, sample-level JSON files were merged before downstream parsing. The resulting SierraPy JSON output was then parsed by HIV-DRLink_github.pl, which groups sequences by shared DRM pattern and writes per-sample DRM summaries. For RT, the script reports predefined major RT drug-resistance mutations and excludes sequences containing only “other” mutations from further DRM-pattern reporting. Output tables include the DRM pattern, the number of sequences with the same pattern, and the corresponding DRM-pattern percentage. For each sample, DRM-pattern frequency was calculated as the number of sequences assigned to that pattern divided by the total number of input sequences for that sample. Accordingly, the summed frequency of all reported DRM patterns reflects the proportion of sample sequences containing one or more reported DRMs, whereas the remainder represents sequences without reported DRMs.

Final outputs included DRM.xlsx, containing sample-level DRM statistics and per-sample DRM pattern sheets, and freq.xlsx, containing barcode statistics and allele-frequency summaries. For the downstream analyses in this study, reported DRM-pattern frequencies were interpreted as sequence-level support for reported DRM patterns. These values should not be interpreted as direct measures of total recovered UMI counts or total template sampling depth.

## Phylogenetic Analysis

Consensus sequences were aligned using the MAFFT web server (<https://mafft.cbrc.jp/>) (3). A maximum likelihood phylogenetic tree was reconstructed to confirm HIV-1 subtype C assignment using the IQ-TREE web server (<http://iqtree.cibiv.univie.ac.at/>) (4). The generalized time reversible model with a proportion of invariable sites and a gamma distribution (GTR + I + G) was selected as the best-fit substitution model using the built-in model selection tool in IQ-TREE, utilizing 4 gamma rate categories. Reference sequences representing major HIV-1 subtypes, including Subtype C, were obtained from the Los Alamos National Laboratory (LANL) HIV Sequence Database (<https://www.hiv.lanl.gov/>) and named using the format 'Subtype_CountryCode.GenBankAccession'. Branch support was assessed using 1, 000 bootstrap replicates. The final tree was rooted and visualized using FigTree v1.4.4.

# Supplementary Results

## Diverse range of mutational patterns observed by NGS-Primer ID across all participants.

A diverse range of mutational patterns were observed within each participant (S3 Fig). The multiple colours within each stacked bar plot represent the proportions of reported DRM-containing and non-DRM patterns identified in each sample. In each participant we observed mutational patterns either in combination with other mutations (linked) or as a single mutation (unlinked). Reported DRM patterns present at frequencies ≤5% of total input sequences are grouped and shown in dark grey for each participant. Low-frequency reported DRM patterns contributed to the overall resistance-pattern profile in each participant and in the presence of majority mutations were associated with high-level resistance to NNRTIs (EFV and NVP) and NRTIs (3TC and FTC). A wide diversity of reported patterns was observed within participants, and approximately one-third of participants had a diverse group of very low-frequency reported DRM patterns (each present at <5% of total input sequences) contributing substantially to the reported pattern profile in those participants.

The presence of any significant majority DRM was associated with VF in 76% (82/108) of the participants. Participants who had at least one reported DRM pattern present at ≥20% of total input sequences had intermediate or greater ART resistance. In those participants with <2 active drugs due to majority DRMs, the risk of VF was greater compared with those with 3 active drugs. Low-frequency reported DRM patterns were present in a large proportion of the participants (96%; 104/108). There were 21 more participants (19%; 21/108) with similar Sanger sequencing susceptibility patterns to participant 103 (GSS=3) where NGS-Primer ID uncovered a substantial proportion of resistant variants undetected by Sanger sequencing. The distribution of DRMs (majority or minority, linked or unlinked) in the remaining participants (78%; 84/108) was characterized and found to be similar to participant 262 (GSS ≤2).

The most common low-frequency reported DRM patterns per drug class were NRTI (M184V, K65R, D67N, L74I, K70E, L210W) and NNRTI (K103N/S, K101E, V106M, G190A/E, Y181C, Y115F, Y188L, V108I, P225H, E138A) mutations. The mutations observed at higher sequence-supported frequencies were also present in various combinations at a minority level (≤5%) further contributing to the diverse range of mutational patterns in each participant. Participants failing EFV-based ART (EFV, FTC and TDF) with dual drug class (NRTI and NNRTI) resistance would have essentially continued treatment (with LPV/r, 3TC, and AZT) on a compromised AZT-3TC backbone due to the presence of TAMs (D67N, L74I, K70E, L210W) and M184V mutation.

The most frequently observed major NRTI mutations observed in our participants were M184V (98%), K65R (51%), type-2 TAMs (D67N (32%), K70R (28%), L74I (19%), and K219Q/R (15%) and the type-1 TAM L210W (S1 Table). The most frequently observed major NNRTI mutations observed in our participants were K103N (97%) followed by V106M (85%), G190A/E (72%), K101E (42%), Y181C (32%), Y188L (28%), Y115F (14%), P225H (12%), V108I (11%) and E138A/K (8%) (S1 Table). The presence of non-polymorphic mutations (V106M and G190A) conferred high-level resistance to NVP and EFV (due to V106M), and intermediate resistance to EFV due to (G190A). There were no significant reductions in susceptibility to other NNRTIs such as rilpivirine (RPV), etravirine (ETR) and doravirine (DOR). The overall proportion of participants with ETR-associated mutations, i.e., L100I, K101P, Y181CIV, G190E, E138A/K/Q, and M230L was 18% (20/107) conferring intermediate to high-level etravirine (ETR).

## Characterization of Resistant and Susceptible variants using weighted GSS by NGS-Primer ID

Weighted genotypic susceptibility scores (wGSS) were calculated for each participant from the reported DRM patterns and their sequence-supported frequencies within the sample. We observed a wide range of resistance patterns with wGSS scores widely distributed across the reported patterns in each participant (Fig 2). The broad distribution of wGSS across the reported patterns within each participant illustrates substantial within-sample resistance diversity not captured by Sanger sequencing. The heatmaps reveal substantial within-sample diversity and an unexpectedly broad distribution of resistance patterns not captured by Sanger sequencing. Sanger sequencing results (indicated by blue dashed box) largely underestimated the amount of resistance in 20% (22/108) of the participants with a GSS of 3, whereas NGS-Primer ID showed a wide distribution of wGSS scores representing resistant and susceptible reported patterns in each of these participants. The underlying diversity in wGSS scores by NGS-Primer ID is impressive, even when Sanger sequencing only captured the majority of the resistance mutations observed (Fig 2). NGS-Primer ID was able to detect a proportion of susceptible patterns which may have been overestimated by traditional NGS-Primer IDs.

# Supplementary Figures

## S1 Fig. Maximum likelihood phylogenetic tree

Maximum-likelihood phylogenetic tree including all sequences from participants in this study together with HIV-1 reference sequences in a rectangular layout. The tree was inferred using the generalized time reversible model with a proportion of invariant sites and gamma-distributed rate variation among sites (GTR + I + G). Branch support was assessed with 1, 000 bootstrap replicates. Branch lengths represent the number of nucleotide substitutions per site (scale bar = 0.03).

## S2 Fig. Schematic illustration of genotypic susceptibility score (GSS) estimation from different sequencing approaches


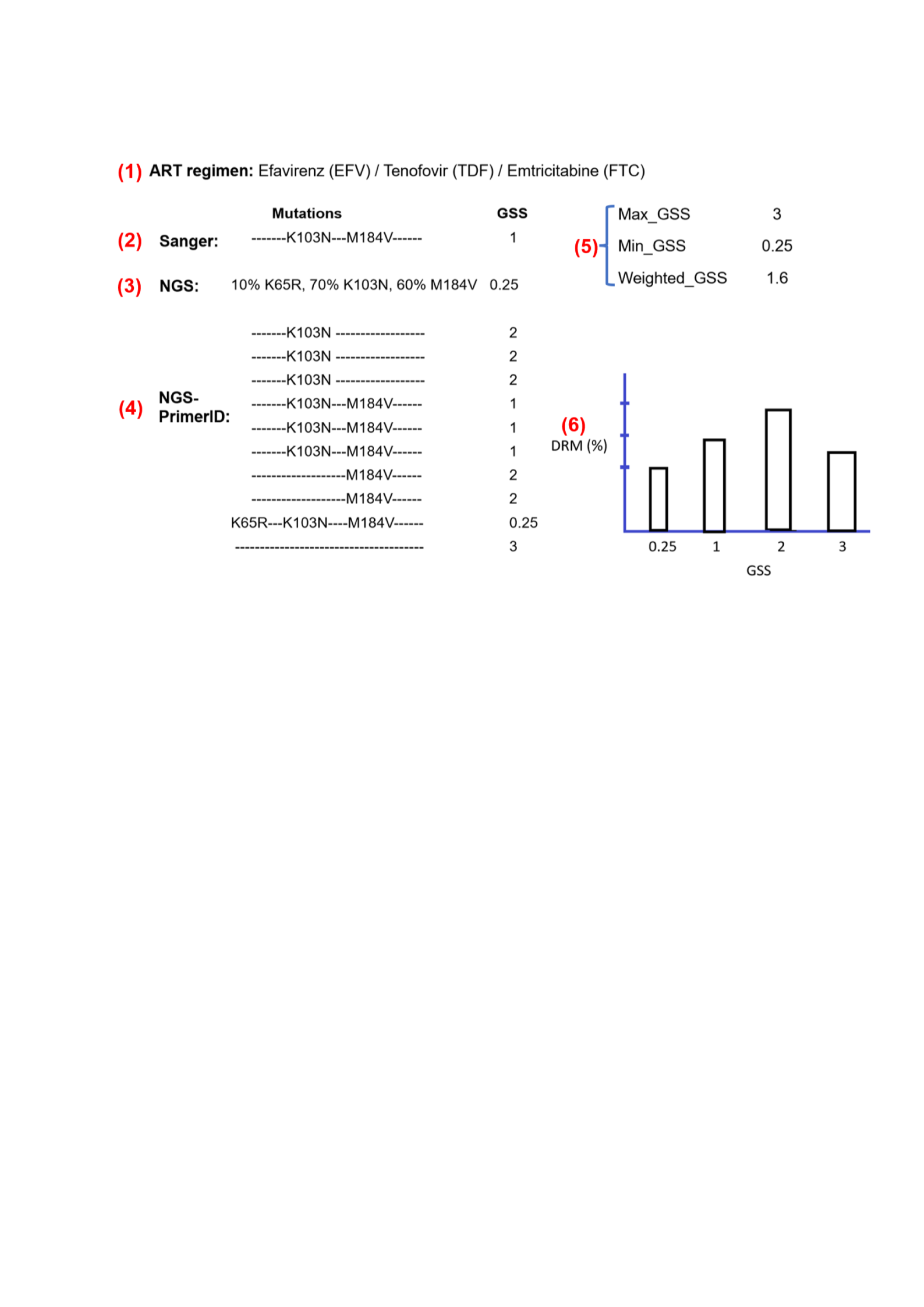


The figure shows a hypothetical participant receiving an antiretroviral regimen consisting of efavirenz (EFV), tenofovir (TDF), and emtricitabine (FTC) (1). Example outputs are shown for Sanger sequencing (2), next-generation sequencing (NGS) (3), and NGS-based ultrasensitive single-genome sequencing with primer identifiers (NGS-PrimerID) (4). For each approach, detected drug resistance mutations are used to derive regimen-level GSS values. The schematic also illustrates summary GSS metrics derived from variant-level data, including maximum GSS, minimum GSS, and weighted GSS (5), and a conceptual distribution of GSS values across detected variants (6).

##
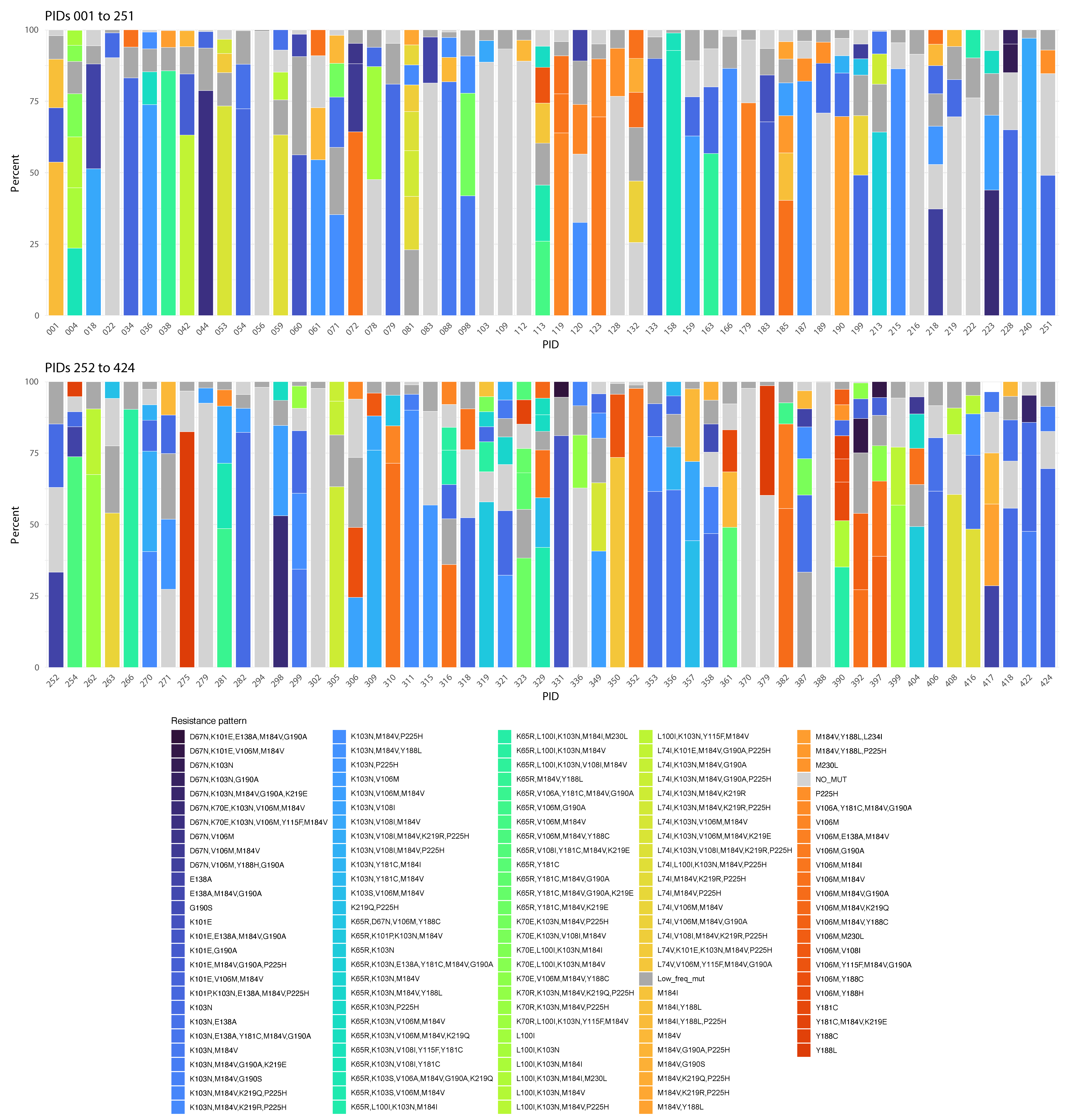
S3 Fig. Distribution of viral mutational patterns across the study cohort

Each stacked bar plot corresponds to an individual participant, and the colors represent specific mutational patterns. Pattern frequencies were calculated from the number of sequences assigned to each pattern relative to the total number of input sequences in the sample. Reported DRM patterns present at frequencies ≤5% are shown in dark grey.

##
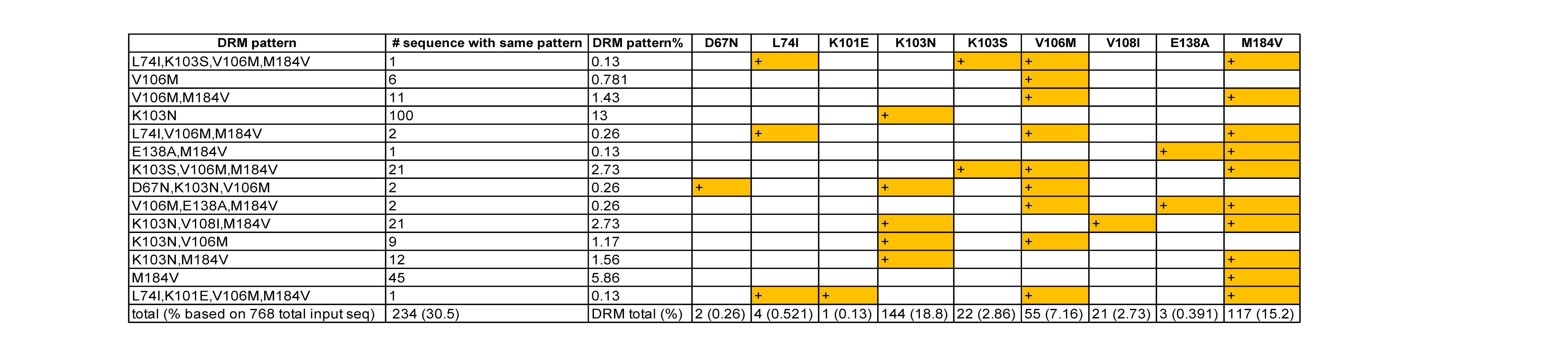
S4 Fig. Detailed mapping of resistance patterns in PID 219

This figure illustrates a specific case where conventional Sanger sequencing underestimated the complexity of the reported resistance-pattern landscape in the sample. The first column identifies distinct mutational patterns, clarifying whether resistance markers are physically linked on individual viral genomes. The second and third columns quantify these patterns by listing the total number of sequences identified and their corresponding percentages within the specimen. Subsequent columns denote the presence of specific drug resistance mutations, which are highlighted with orange boxes. The final row provides the cumulative frequency of each individual mutation relative to the total number of input sequences in the sample.

##
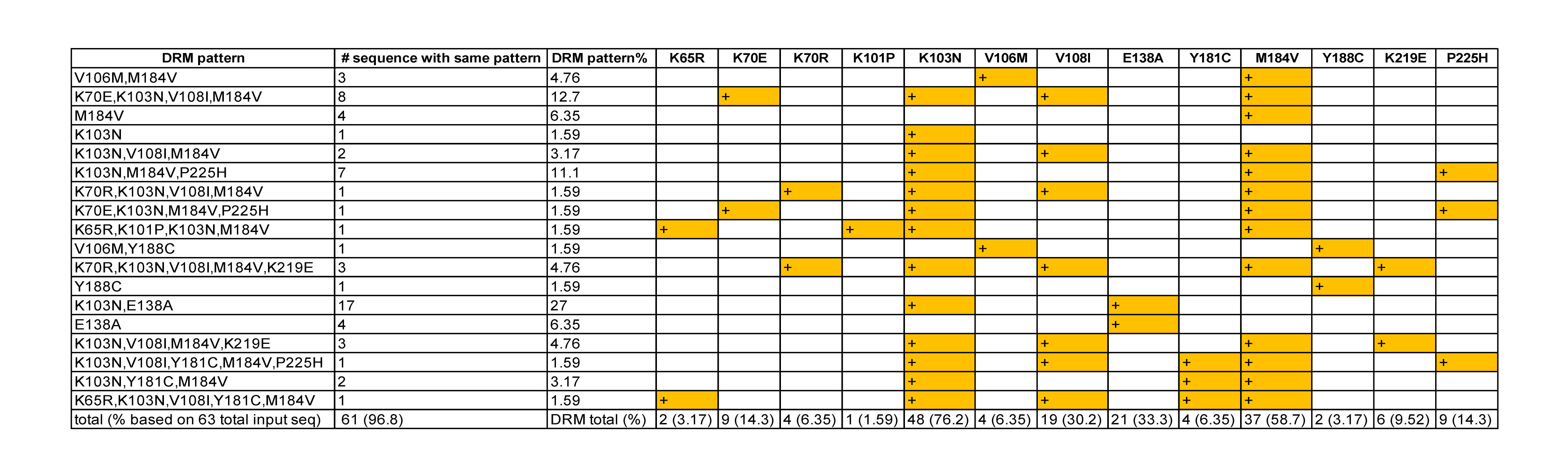
S5 Fig. Detailed mapping of resistance patterns in PID 387

This figure illustrates a specific case where conventional Sanger sequencing overestimated the complexity of the reported resistance-pattern landscape in the sample. The first column identifies distinct mutational patterns, clarifying whether resistance markers are physically linked on individual viral genomes. The second and third columns quantify these patterns by listing the total number of sequences identified and their corresponding percentages within the specimen. Subsequent columns denote the presence of specific drug resistance mutations, which are highlighted with orange boxes. The final row provides the cumulative frequency of each individual mutation relative to the total number of input sequences in the sample.

# Supplementary Tables

## S1 Table: Sample-level clinical, Sanger genotypic susceptibility, and NGS-Primer ID pattern summary metrics at HIV viral load time point 1.

| **Patient_ID** | **Sex (M/F)** | **Age** | **CD4_**  **Baseline** | **Regimens** | **HIVVL Baseline** | **NRTI (SS)** | **NNRTI (SS)** | **FTC_GSS_score** | **TDF_GSS_score** | **EFV_GSS_score** | **Sanger_Gross_GSS_score** | **Total number of patterns identified, n** | **Total number of sequences across all patterns, n** |
| --- | --- | --- | --- | --- | --- | --- | --- | --- | --- | --- | --- | --- | --- |
| 1 | F | 37 | 354 | EFV, FTC, TDF | 175388 |  |  | 1 | 1 | 0 | 3 | 16 | 2153 |
| 22 | M | 29 | 36 | EFV, FTC, TDF | 6537 |  |  | 1 | 1 | 0 | 3 | 3 | 92 |
| 56 | M | 36 | 35 | EFV, FTC, TDF | 612883 |  |  | 1 | 1 | 0 | 3 | 12 | 8956 |
| 78 | M | 30 | 345 | EFV, FTC, TDF | 107470 |  |  | 1 | 1 | 0 | 3 | 9 | 651 |
| 83 | F | 33 | 159 | EFV, FTC, TDF | 226000 |  | E138A | 1 | 1 | 1 | 3 | 7 | 553 |
| 109 | M | 18 | 541 | EFV, FTC, TDF | 4459 |  |  | 1 | 1 | 0 | 3 | 4 | 283 |
| 183 | F | 25 | 507 | EFV, FTC, TDF | 4730 |  |  | 1 | 1 | 0 | 3 | 7 | 183 |
| 187 | F | 42 | 1275 | EFV, FTC, TDF | 108000 |  |  | 1 | 1 | 0 | 3 | 5 | 50 |
| 279 | M | 31 | 273 | EFV, FTC, TDF | 583558 |  |  | 1 | 1 | 0 | 3 | 15 | 3601 |
| 370 | F | 40 | 611 | EFV, FTC, TDF | 7192 |  |  | 1 | 1 | 0 | 3 | 4 | 508 |
| 379 | F | 22 | 753 | EFV, FTC, TDF | 23134 |  |  | 1 | 1 | 0 | 3 | 10 | 1069 |
| 18 | F | 28 | 101 | EFV, FTC, TDF | 212220 | M184V | K103N, V108I | 0 | 1 | 0 | 1 | 10 | 502 |
| 34 | F | 56 | 74 | EFV, FTC, TDF | 64138 | M184V | K101P, K103N, E138A, P225H | 0 | 1 | 0 | 1 | 17 | 297 |
| 36 | M | 60 | 21 | EFV, FTC, TDF | 69182 | M184MV | K103KN, V106VM | 0 | 1 | 0 | 1 | 14 | 374 |
| 42 | F | 36 | 398 | EFV, FTC, TDF | 12681 | L74I, M184V | K101E, G190A, P225H | 0 | 1 | 0 | 1 | 19 | 608 |
| 53 | M | 40 | 331 | EFV, FTC, TDF | 2074 | M41L, L74I, M184V, K219KDEN | K103N, V106M | 0 | 1 | 0 | 1 | 10 | 60 |
| 60 | F | 29 | 492 | EFV, FTC, TDF | 6819 | D67G | V106M, Y188YC | 1 | 1 | 0 | 2 | 17 | 64 |
| 61 | F | 18 | 331 | EFV, FTC, TDF | 2445 |  | K103N, P225H | 1 | 1 | 0 | 2 | 4 | 22 |
| 72 | M | 39 | 476 | EFV, FTC, TDF | 4544 | D67DN, M184V | V106M, F227L | 0 | 1 | 0 | 1 | 5 | 42 |
| 79 | M | 53 | 251 | EFV, FTC, TDF | 1558 |  | K103N | 1 | 1 | 0 | 2 | 5 | 21 |
| 88 | M | 45 | 624 | EFV, FTC, TDF | 69998 | M184V | K103N | 0 | 1 | 0 | 1 | 7 | 517 |
| 98 | M | 25 | 355 | EFV, FTC, TDF | 40470 | L74LI, M184V | A98G, K103N, E138G, P225H | 0 | 1 | 0 | 1 | 11 | 739 |
| 103 | M | 43 | 219 | EFV, FTC, TDF | 271201 |  |  | 1 | 1 | 0 | 3 | 4 | 53 |
| 112 | M | 44 | 139 | EFV, FTC, TDF | 1834 |  |  | 1 | 1 | 0 | 3 | 5 | 82 |
| 113 | F | 39 | 235 | EFV, FTC, TDF | 9807 |  |  | 1 | 1 | 0 | 3 | 25 | 799 |
| 119 | M | 50 | 113 | EFV, FTC, TDF | 2177293 | M184MV | V106M, V179VD | 0 | 1 | 0 | 1 | 24 | 4837 |
| 123 | F | 24 | 417 | EFV, FTC, TDF | 228387 |  | V106M, G190GA | 1 | 1 | 0 | 2 | 29 | 7303 |
| 128 | M | 51 | 281 | EFV, FTC, TDF | 33332 |  |  | 1 | 1 | 0 | 3 | 8 | 185 |
| 132 | F | 36 | 433 | EFV, FTC, TDF | 12313 | L74LI, M184V | V106M, G190A | 0 | 1 | 0 | 1 | 19 | 219 |
| 133 | M | 34 | 66 | EFV, FTC, TDF | 37493 |  | K103N | 1 | 1 | 0 | 2 | 17 | 1610 |
| 159 | F | 44 | 225 | EFV, FTC, TDF | 799000 |  | K103N, V106M | 1 | 1 | 0 | 2 | 33 | 3704 |
| 166 | F | 33 | 234 | EFV, FTC, TDF | 3294 | M184V | K103N, P225H | 0 | 1 | 0 | 1 | 13 | 303 |
| 179 | M | 38 | 248 | EFV, FTC, TDF | 73400 | M184V | V106M, E138A, V179D | 0 | 1 | 0 | 1 | 11 | 1487 |
| 185 | M | 34 | 395 | EFV, FTC, TDF | 12200 | M184V | K103KN, V106M, V179VD | 0 | 1 | 0 | 1 | 20 | 409 |
| 189 | M | 32 | 698 | EFV, FTC, TDF | 51000 |  |  | 1 | 1 | 0 | 3 | 7 | 460 |
| 190 | M | 49 | 200 | EFV, FTC, TDF | 1620 | T69D, M184V | V179D, Y188L | 0 | 1 | 0 | 1 | 5 | 33 |
| 199 | F | 34 | 261 | EFV, FTC, TDF | 22600 |  | K103N | 1 | 1 | 0 | 2 | 21 | 523 |
| 215 | M | 42 | 538 | EFV, FTC, TDF | 2690 | M184V | K103N, P225H | 0 | 1 | 0 | 1 | 3 | 22 |
| 216 | F | 45 | 319 | EFV, FTC, TDF | 47100 |  |  | 1 | 1 | 0 | 3 | 13 | 397 |
| 218 | F | 32 | 1200 | EFV, FTC, TDF | 11100 | D67N | V106M, Y188H, G190A | 1 | 1 | 0 | 2 | 21 | 335 |
| 219 | M | 56 | 213 | EFV, FTC, TDF | 123000 |  |  | 1 | 1 | 0 | 3 | 15 | 768 |
| 228 | F | 28 | 525 | EFV, FTC, TDF | 48000 | D67N | K103N, G190A | 1 | 1 | 0 | 2 | 4 | 20 |
| 240 | F | 45 | 423 | EFV, FTC, TDF | 1020 | M184V | K103N, V108I | 0 | 1 | 0 | 1 | 3 | 34 |
| 251 | F | 33 | 307 | EFV, FTC, TDF | 14300 |  | K103N | 1 | 1 | 0 | 2 | 6 | 267 |
| 254 | F | 34 | 93 | EFV, FTC, TDF | 2420 | NONE | V179D | 1 | 1 | 0.75 | 2.75 | 5 | 19 |
| 263 | F | 33 | 269 | EFV, FTC, TDF | 8980 | L74I, M184V | V106M, H221Y, F227L | 0 | 1 | 0 | 1 | 19 | 374 |
| 271 | M | 54 | 98 | EFV, FTC, TDF | 38424 |  | K103N, P225PH | 1 | 1 | 0 | 2 | 19 | 274 |
| 275 | M | 39 | 81 | EFV, FTC, TDF | 55871 |  | Y188L | 1 | 1 | 0 | 2 | 12 | 811 |
| 282 | F | 23 | 4 | EFV, FTC, TDF | 25414 |  | K103N, V106VM | 1 | 1 | 0 | 2 | 11 | 1155 |
| 294 | M | 41 | 61 | EFV, FTC, TDF | 87843 |  |  | 1 | 1 | 0 | 3 | 22 | 3924 |
| 302 | M | 52 | 613 | EFV, FTC, TDF | 1695 |  |  | 1 | 1 | 0 | 3 | 2 | 42 |
| 306 | F | 30 | 701 | EFV, FTC, TDF | 3856 |  | K103KN, V106M, Y188C | 1 | 1 | 0 | 2 | 12 | 49 |
| 309 | F | 24 | 366 | EFV, FTC, TDF | 1270 |  | K103KN, V108VI | 1 | 1 | 0 | 2 | 5 | 25 |
| 310 | M | 40 | 747 | EFV, FTC, TDF | 5103 | M184V | V106M, F227L | 0 | 1 | 0 | 1 | 6 | 84 |
| 311 | M | 31 | 364 | EFV, FTC, TDF | 2508 | M184V | K103N, P225H | 0 | 1 | 0 | 1 | 6 | 90 |
| 315 | F | 44 | 154 | EFV, FTC, TDF | 10117 |  | K103N, P225PH | 1 | 1 | 0 | 2 | 12 | 537 |
| 316 | F | 18 | 313 | EFV, FTC, TDF | 3403 | M184V | K103N, P225H | 0 | 1 | 0 | 1 | 10 | 25 |
| 331 | M | 30 | 563 | EFV, FTC, TDF | 5577 | M184V | E138A | 0 | 1 | 1 | 2 | 17 | 201 |
| 336 | F | 31 | 413 | EFV, FTC, TDF | 25787 | M41L, K70R, M184V, K219Q | K103N, P225H | 0 | 1 | 0 | 1 | 31 | 1234 |
| 349 | M | 36 | 56 | EFV, FTC, TDF | 43022 | L74LI, M184V | K103N, V106M | 0 | 1 | 0 | 1 | 23 | 848 |
| 350 | F | 50 | 30 | EFV, FTC, TDF | 250976 | L74V, Y115F, M184V | V106M, V179D, G190A | 0 | 1 | 0 | 1 | 21 | 1825 |
| 352 | F | 53 | 441 | EFV, FTC, TDF | 2261 | M184V | V106M, V179D | 0 | 1 | 0 | 1 | 3 | 84 |
| 356 | M | 54 | 547 | EFV, FTC, TDF | 1720 | M184V | K103N | 0 | 1 | 0 | 1 | 13 | 140 |
| 357 | M | 49 | 44 | EFV, FTC, TDF | 30714 | M184V | K103NS, V106VM, V108VI | 0 | 1 | 0 | 1 | 7 | 397 |
| 358 | F | 31 | 24 | EFV, FTC, TDF | 681271 |  | K103N | 1 | 1 | 0 | 2 | 22 | 1492 |
| 361 | M | 44 | 332 | EFV, FTC, TDF | 7219 | M184V | K103N, V108I, P225H | 0 | 1 | 0 | 1 | 17 | 1209 |
| 382 | M | 25 | 494 | EFV, FTC, TDF | 32200 |  | V106M, G190A | 1 | 1 | 0 | 2 | 6 | 27 |
| 388 | M | 53 | 477 | EFV, FTC, TDF | 53274 |  |  | 1 | 1 | 0 | 3 | 3 | 1057 |
| 390 | M | 43 | 57 | EFV, FTC, TDF | 35177 |  | V106M, Y188C | 1 | 1 | 0 | 2 | 10 | 37 |
| 392 | F | 39 | 44 | EFV, FTC, TDF | 5389 | D67DGNS, M184V | K101KE, V106M, Y188YC, F227L | 0 | 1 | 0 | 1 | 32 | 449 |
| 397 | M | 23 | 604 | EFV, FTC, TDF | 2898 | M184V, D67N | K101P, K103N, E138A | 0 | 1 | 0 | 1 | 31 | 914 |
| 399 | F | 41 | 502 | EFV, FTC, TDF | 6088 |  |  | 1 | 1 | 0 | 3 | 18 | 523 |
| 404 | F | 31 | 58 | EFV, FTC, TDF | 29574 |  | K103N | 1 | 1 | 0 | 2 | 14 | 150 |
| 406 | F | 50 | 289 | EFV, FTC, TDF | 43700 |  | V106M, Y188H | 1 | 1 | 0 | 2 | 9 | 107 |
| 408 | F | 40 | 351 | EFV, FTC, TDF | 20351 |  | K103N | 1 | 1 | 0 | 2 | 9 | 119 |
| 417 | F | 21 | 388 | EFV, FTC, TDF | 67834 |  | K103N | 1 | 1 | 0 | 2 | 6 | 28 |
| 418 | F | 64 | 771 | EFV, FTC, TDF | 1140 |  | K103N | 1 | 1 | 0 | 2 | 8 | 97 |
| 422 | M | 36 | 123 | EFV, FTC, TDF | 1292 | D67D/N, M184V, K219E | K103N, G109A | 0 | 1 | 0 | 1 | 4 | 21 |
| 424 | F | 29 | 347 | EFV, FTC, TDF | 69737 |  | K103N | 1 | 1 | 0 | 2 | 5 | 23 |
| 353 | M | 26 | 1234 | EFV, FTC, TDF | 9440 | M184V | K103N, P225H | 0 | 1 | 0 | 1 | 6 | 26 |
| 416 | M | 28 | 23 | EFV, FTC, TDF | 205571 | M184V, L74I | L100I, K103N, P225H | 0 | 1 | 0 | 1 | 8 | 62 |
| 299 | M | 46 | 219 | EFV, FTC, TDF | 1043 | M184V | K103N, P225H | 0 | 1 | 0 | 1 | 6 | 64 |
| 318 | F | 41 | 204 | EFV, FTC, TDF | 10038 | D67N, M184V, K219E | V106A | 0 | 1 | 0.25 | 1.25 | 5 | 21 |
| 4 | M | 31 | 280 | EFV, FTC, TDF | 157415 | D67G, K70KE, M184V | L100I, K103N | 0 | 0.5 | 0 | 0.5 | 24 | 550 |
| 38 | F | 49 | 379 | EFV, FTC, TDF | 23787 | K65R, M184V | Y188L | 0 | 0.25 | 0 | 0.25 | 17 | 745 |
| 44 | M | 33 | 98 | EFV, FTC, TDF | 18635 | D67N, K70E, Y115F, M184V | K103N, V106M | 0 | 0.25 | 0 | 0.25 | 27 | 625 |
| 222 | F | 46 | 146 | EFV, FTC, TDF | 1450 | A62AV, K65R, M184V | L100I, K103N | 0 | 0.25 | 0 | 0.25 | 14 | 193 |
| 223 | F | 30 | 2 | EFV, FTC, TDF | 634000 | M184V | Y188L | 0 | 1 | 0 | 1 | 18 | 385 |
| 213 | M | 47 | 55 | EFV, FTC, TDF | 305000 | K65R, M184V | K101H, K103N, E138A, Y181C, G190A, H221Y | 0 | 0.25 | 0 | 0.25 | 36 | 886 |
| 262 | F | 31 | 28 | EFV, FTC, TDF | 198633 | K65N, K70R, Y115F, M184V | L100I, K103N, H221Y | 0 | 0.25 | 0 | 0.25 | 27 | 3048 |
| 266 | M | 42 | 16 | EFV, FTC, TDF | 999 | A62AV, K65R, M184V | L100I, K103N | 0 | 0.25 | 0 | 0.25 | 18 | 1343 |
| 270 | M | 38 | 726 | EFV, FTC, TDF | 4325 | K70N, M184V, K219R | K103N, V108I, P225H | 0 | 0.5 | 0 | 0.5 | 6 | 37 |
| 281 | F | 30 | 328 | EFV, FTC, TDF | 2459 | K65R, M184V, K219Q | K103GS, V106A, G190A | 0 | 0.25 | 0 | 0.25 | 6 | 35 |
| 298 | M | 41 | 457 | EFV, FTC, TDF | 8823 | A62AV, K65KR, D67DN, K70KE, L74LI, M184V | K103N, V106M | 0 | 0 | 0 | 0 | 16 | 671 |
| 305 | M | 29 | 290 | EFV, FTC, TDF | 20469 | K70Q, L74I, M184V, K219KR | A98AG, K103N, G190GA, F227L | 0 | 0.5 | 0 | 0.5 | 20 | 204 |
| 323 | F | 41 | 280 | EFV, FTC, TDF | 14087 | K65R, M184V, K219E | Y181C, G190GA | 0 | 0.25 | 0 | 0.25 | 14 | 47 |
| 329 | M | 39 | 278 | EFV, FTC, TDF | 14453 | A62V, K65R, K70KT, M184V | K103NS, V106M | 0 | 0 | 0 | 0 | 12 | 138 |
| 71 | F | 41 | 343 | EFV, FTC, TDF | 8980 | K70KE, M184V | K103N, P225H | 0 | 0.5 | 0 | 0.5 | 16 | 51 |
| 158 | F | 58 | 386 | EFV, FTC, TDF | 5800 | K65R, Y115YF | K103N, V108I, Y181C, H221HY | 0.5 | 0 | 0 | 0.5 | 7 | 620 |
| 163 | F | 42 | 257 | EFV, FTC, TDF | 2280 | K65R, M184V | A98G, L100I, K103N, V108I | 0 | 0.25 | 0 | 0.25 | 5 | 30 |
| 54 | F | 25 | 8 | EFV, FTC, TDF | 161582 | K65R, T69Deletion | V106M, Y181C, H221Y | 0.25 | 0 | 0 | 0.25 | 14 | 315 |
| 59 | M | 32 | 629 | EFV, FTC, TDF | 6931 | K65R, Y115F | K103N, V106M | 0.5 | 0 | 0 | 0.5 | 16 | 155 |
| 81 | M | 39 | 286 | EFV, FTC, TDF | 3696 | K70KN, L74I, Y115Y, M184V, K219R | A98G, K103N, V108VI, P225H | 0 | 0.25 | 0 | 0.25 | 38 | 1072 |
| 120 | F | 41 | 223 | EFV, FTC, TDF | 40389 | K65R, T69del | V106M, Y181C, Y188C | 0.25 | 0 | 0 | 0.25 | 9 | 46 |
| 252 | F | 48 | 55 | EFV, FTC, TDF | 2199 | K65R, M184V, K219E | K103N, V108I, M230L, L234I | 0 | 0.25 | 0 | 0.25 | 7 | 27 |
| 319 | F | 34 | 235 | EFV, FTC, TDF | 1386 | A62AV, K65R, M184V | K101P, K103N, V179L | 0 | 0.25 | 0 | 0.25 | 7 | 19 |
| 321 | M | 34 | 112 | EFV, FTC, TDF | 2483 | K65R, M184V | K103N, Y188L | 0 | 0.25 | 0 | 0.25 | 8 | 31 |
| 387 | F | 26 | 87 | EFV, FTC, TDF | 3139 | D67DN, K70KEGR, M184V, T215I, K219E | K103N, V108I | 0 | 0.25 | 0 | 0.25 | 19 | 63 |

**PID,** Participant Identifier**; HIVVL,** HIV viral load**; mL;** millilitre**; mm^3^,** cubic millimetres**; GSS,** Genotypic Susceptibility Scores**; Efavirenz,** EFV**); Emtricitabine,** FTC**; Tenofovir,** TDF**; CD4,** Cluster of Differentiation 4**; NRTI,** Nucleoside/Nucleotide Reverse Transcriptase Inhibitor**; NNRTI,** Non-Nucleoside Reverse Transcriptase Inhibitor**.** Pattern counts include both DRM-containing patterns and the pattern representing sequences without detected DRMs.

## S2 Table: Genotypic Susceptibility Scores with corresponding susceptibility levels

| **Total Penalty Score** | **Susceptibility Level** | **Genotypic Susceptibility**  **Scores** |
| --- | --- | --- |
| <10 | Susceptible | 1 |
| 10 to <15 | Potential low-level resistance | 0.75 |
| 15 to <30 | Low-level resistance | 0.5 |
| 30 to <60 | Intermediate resistance | 0.25 |
| ≥60 | High-level resistance | 0 |

## S3 Table: Selection Criteria for Plasma specimens GSS ≥1

| **Selection Criteria** | **SOC** | **RT** | **Total** |
| --- | --- | --- | --- |
| Patient sequences (TP 1) | 210 | 170 | 380 |
| Patient sequences with GSS ≥1(TP 1) | 87 | 83 | 170 |
| Patients with GSS ≥1 (TP 1), maintained on first-line ART with VF at (TP 3) (HIVVL ≥1000 cp/ml) | 62 | 5 | 67 |
| Patients with GSS ≥1, maintained on first-line ART, without VF at Time Point 3 (HIVVL<1000 cp/ml) | 20 | 6 | 26 |

**VF,** Virologic Failure**; GSS,** Genotypic Susceptibility Scores**; SOC,** Standard of Care**; RT,** Resistance Testing**; VL,** Viral load**; ART,** Antiretroviral Therapy**; TP,** Timepoint

## S4 Table: Selection criteria used for Plasma specimens with GSS <1

| **Selection Criteria** | **SOC** |
| --- | --- |
| Patient sequences (TP 1) | 210 |
| Patient sequences with GSS <1 (TP 1) | 101 |
| Patients with GSS <1 (TP 1) without VF at TP 2  (HIVVL <1000 cp/ml) | 14 |
| Patients with GSS <1 with VF at TP 2 (HIVVL≥1000 cp/ml). | 14 |

## S5 Table: Participant specimens selected from the REVAMP Study for NGS-Primer ID

| **Study Arm** | **Number of specimens** | **Time Point** | **Sanger-based GSS** | **ART Maintained or Switched** | **Assay** | **Analysis** |
| --- | --- | --- | --- | --- | --- | --- |
| RT and SOC | 93 | 1 | ≥1 | Maintained on  first-line ART | NGS-Primer ID | NGS-derived GSS and  Resistance Linkage |
| SOC | 14 | 1 | <1 |  |  |  |

**NGS-Primer ID,** Next Generation Sequencing-Primer Identifier**.**

## S6 Table: cDNA Primers with Primer ID tags used for reverse transcription

| **Primer Name** | **Primer Sequence (5’-3’)** |
| --- | --- |
| **GSPID-3271R C** | GGTATCGAAGTCATCCTGCTAGNNNNNNNNNNACTGTCCATTTGTCAGGATG |
| **GSPID-3271R C (Tvar)** | GGTATCGAAGTCATCCTGCTAGNNNNNNNNNNACTGTCCATTT**A**TCAGGATG |
| **GSPID-3271R C (Gvar)** | GGTATCGAAGTCATCCTGCTAGNNNNNNNNNNACTGTCCA**C**TTGTCAGGATG |
| **GSPID-3271R C (Cvar)** | GGTATCGAAGTCATCCTGCTAGNNNNNNNNNNACTGTCCATTTGTCAGG**G**TG |
| **GSPID-3271R C (ACvar):** | GGTATCGAAGTCATCCTGCTAGNNNNNNNNNNACTGTCCATTTG**GT**AGGATG |

Primer sequence consists of priming region (Green), 10bp Primer ID tag (Blue), gene specific region (Orange) and base pairs that are different in the variant specific primers compared to the GSPID-3271R (red bold)

## S7 Table: Complementary DNA synthesis Master Mix 1

| **Reagent** | |  | **Volume per reaction (µl)** | |
| --- | --- | --- | --- | --- |
| **Nuclease-free Water** | |  | | **0.0** |
| **Deoxynucleotide triphosphate (dNTP)** | |  | | 2.5 |
| **GSPID Primer (5µM)** | |  | | 2.5 |
| **RNA** | |  | | **20** |
| **Total Volume** | |  | | 25 |
| **Thermocycling Conditions** | | | | |
|  | **Temperature (^o^C)** | **Time** | | **Cycle(s)** |
| **Denaturation** | **85** | **10 minutes** | | **1** |
| **Cool** | **Snap-freeze** | **1 minute** | | **-** |

## S8 Table: Complementary DNA synthesis Master Mix 2

| **Reagent** | | | |  | | **Volume per reaction (µl)** | |
| --- | --- | --- | --- | --- | --- | --- | --- |
| Nuclease-free Water | | | |  | | 7.5 | |
| 5x First-strand Buffer | | | |  | | 10.0 | |
| RNase out Inh | | | |  | | 2.5 | |
| DTT (dithiothreitol) | | | |  | | 2.5 | |
| SSIV/ Platinum SuperFi DNA polymerase (2X) | | | |  | | 2.5 | |
| **Total volume** | | | |  | | **25** | |
| **Thermocycling Conditions** | | | | | | | |
|  | **Temperature (^o^C)** | | **Time** | | | | **Cycle(s)** |
|  |  |  |  | |  | |  |
| Denaturation  cDNA Synthesis |  | 85 |  | | 10 minutes | | 1 |
|  |  | 45 |  | | 1 Hour | | 1 |
| Hold |  | 4 |  | | ∞ | | Hold |

## S9 Table: First-Round Master Mix and Conditions

| **Reagent** | |  | **Volume per Reaction (µl)** | |
| --- | --- | --- | --- | --- |
| KAPA HiFi Uracil + (2X) | |  | 12.5 | |
| Nuclease-free Water | |  | | 6 |
| 2589 forward (10µM) | |  | | 0.75 |
| primRegion-R-5Us (10µM) | |  | | 0.75 |
| **Total Volume** | |  | | **20** |
| Template | |  | | **5** |
| **Thermocycling Conditions** | | | | |
|  | **Temperature (^o^C)** | **Time** | | **Cycle(s)** |
| Pre-denaturation | 95 | 3 minutes | | 1 |
| Denaturation | 98 | 15 seconds | | 15 |
| Annealing | 60 | 30 seconds | |  |
| Extension | 72 | 30 seconds | |  |
| Final Extension | 72 | 1 minute | | 1 |
| Hold | 4 | ∞ | | Hold |

## S10 Table: Second-Round Master Mix and Conditions

| **Reagent** | | **Volume per Reaction (µl)** | |  |
| --- | --- | --- | --- | --- |
| KAPA HiFi Uracil + (2X) | | 12.5 | |  |
| Nuclease-free Water | | 6 | |  |
| 2709 forward (10µM) | | 0.75 | |  |
| primRegion-R-5Us (10µM) | | 0.75 | |  |
| **Total Volume** | | **20** | |  |
| Template | | **5µl** | |  |
| **Thermocycling Conditions** | | | | |
|  | **Temperature (^o^C)** | **Time** | **Cycle(s)** | |
| Pre-denaturation | 95 | 3 minutes | 1 | |
| Denaturation | 98 | 15 seconds | 28 | |
| Annealing | 52 | 30 seconds |  |  |
| Extension | 72 | 30 seconds |  |  |
| Final Extension | 72 | 1 minute | 1 | |
| Hold | 4 | ∞ | Hold | |

## S11 Table: cDNA Amplification Primers used in First and Second Round PCR

| **Primer Name** | **Direction** | **Primer Sequence (5’-3’)** |
| --- | --- | --- |
| 2589 FC | Forward | 5’CCAGGAATGGATGGCCCAA3’ |
| 2709 FC | Forward | 5’AAYCCAdUAdUAAYACdUCCARdUATTTG 3’ |
| PrimRegion-R-5Us | Reverse | 5′ GGdUAdUCGAAGdUCAdUCCdUGCTAG 3′ |

**References:**

1. Siedner MJ, Moosa M-YS, McCluskey S, Gilbert RF, Pillay S, Aturinda I, et al. Resistance testing for management of HIV virologic failure in sub-Saharan Africa: an unblinded randomized controlled trial. Annals of Internal Medicine. 2021;174(12):1683-92.

2. Liu TF, Shafer RW. Web resources for HIV type 1 genotypic-resistance test interpretation. Clin Infect Dis. 2006;42(11):1608-18.

3. Katoh K, Rozewicki J, Yamada KD. MAFFT online service: multiple sequence alignment, interactive sequence choice and visualization. Brief Bioinform. 2019;20(4):1160-6.

4. Trifinopoulos J, Nguyen LT, von Haeseler A, Minh BQ. W-IQ-TREE: a fast online phylogenetic tool for maximum likelihood analysis. Nucleic Acids Res. 2016;44(W1):W232-5.
